# Structural basis for self-assembly of a cytolytic pore lined by protein and lipid

**DOI:** 10.1038/ncomms7337

**Published:** 2015-02-26

**Authors:** Koji Tanaka, Jose M.M. Caaveiro, Koldo Morante, Juan Manuel González-Mañas, Kouhei Tsumoto

**Affiliations:** 1 Department of Chemistry and Biotechnology, School of Engineering, The University of Tokyo, Bunkyo-ku, Tokyo 113-8656, Japan; 2 Department of Bioengineering, School of Engineering, The University of Tokyo, Bunkyo-ku, Tokyo 113-8656, Japan; 3 Department of Biochemistry and Molecular Biology, University of the Basque Country, Lejona, Vizcaya 48940, Spain; 4 Laboratory of Medical Proteomics, Institute of Medical Science, The University of Tokyo, 4-6-1 Shirokanedai, Minato-ku, Tokyo 108-8639, Japan

## Abstract

Pore-forming toxins (PFT) are water-soluble proteins that possess the remarkable ability to self-assemble on the membrane of target cells, where they form pores causing cell damage. Here, we elucidate the mechanism of action of the haemolytic protein fragaceatoxin C (FraC), a α-barrel PFT, by determining the crystal structures of FraC at four different stages of the lytic mechanism, namely the water-soluble state, the monomeric lipid-bound form, an assembly intermediate and the fully assembled transmembrane pore. The structure of the transmembrane pore exhibits a unique architecture composed of both protein and lipids, with some of the lipids lining the pore wall, acting as assembly cofactors. The pore also exhibits lateral fenestrations that expose the hydrophobic core of the membrane to the aqueous environment. The incorporation of lipids from the target membrane within the structure of the pore provides a membrane-specific trigger for the activation of a haemolytic toxin.

## Abstract (web summary)

Actinoporins are water-soluble pore-forming toxins that self-assemble in the membranes of target cells. Here, the authors provide insight into the mechanism of membrane pore formation by solving the structures of several states of the hemolytic protein fragaceatoxin C, including the fully assembled pore.

## Graphical abstract

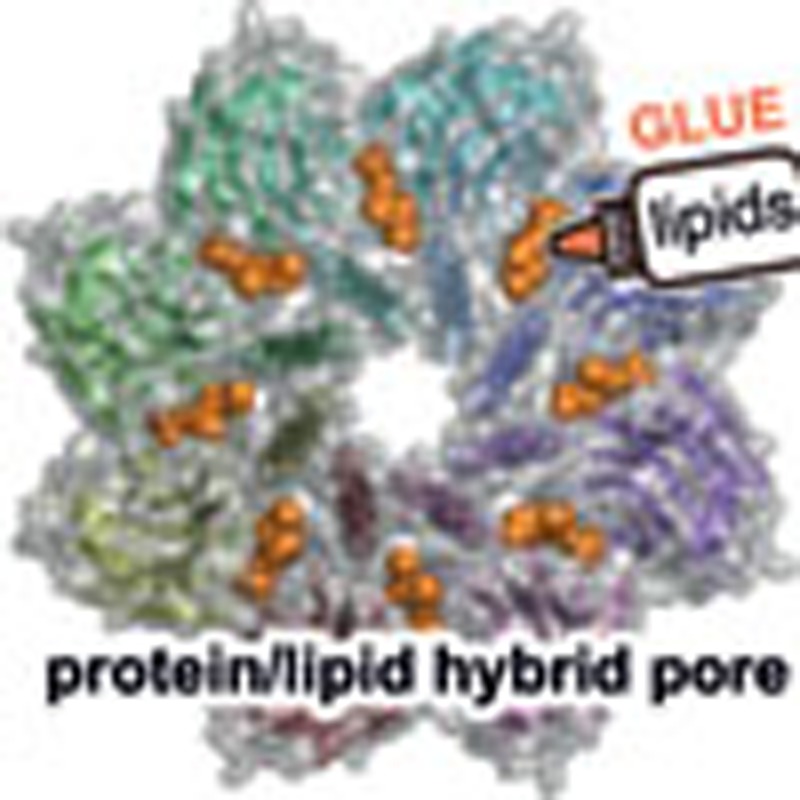

At the cellular and molecular levels, organisms are engaged in a permanent battle for survival requiring specialized offensive and defensive systems. Pore-forming toxins (PFT), exemplified by the membrane attack complex of the immune system of vertebrates, and by some virulence factors of pathogenic bacteria, are key molecular components of these frontline systems^1^,^2^. PFT are water-soluble proteins with the remarkable ability to spontaneously self-assemble into transmembrane pores on the lipid membrane of the target cell, causing cell-damage^3^,^4^. It is assumed that lipids act as binding receptors and provide the hydrophobic environment necessary for the large conformational changes (metamorphosis) of PFTs^5^,^6^. We note, however, that the preparation of the oligomeric pore of PFTs for crystallographic studies does not generally require lipids. Instead, the pore is generated in hydrophobic environments using detergents or alcohols as a surrogate for lipid bilayers^7^,^8^,^9^, with the exception of that of *Vibrio cholerae*^10^.

Fragaceatoxin C (FraC)^11^ is a potent haemolysin of the family of actinoporins, defined as a group of ~20 kDa protein toxins produced by sea anemones. Actinoporins are classified as α-helical PFT (α-PFT) because the transmembrane pore is predicted to form an α-helical barrel^12^,^13^. The lytic activity of actinoporins on biological membranes is enhanced by the lipid sphingomyelin (SM) and by lipid-phase coexistence^14^,^15^,^16^. The simultaneous manifestation of these two features activates the toxin, triggering the formation of aqueous pores followed by widespread cellular damage on susceptible cells. Previously, the structure of the transmembrane pore of actinoporins was studied with electron microscopy (EM), yielding two contrasting models—a 9-mer α-helical-bundle pore^17^, and a tetrameric toroidal pore formed by non-interacting proteins glued together by lipids from the membrane^18^. So far, the crystal structure of the active pore at atomic resolution has been elusive, partially because the active pores of FraC must be obtained in the presence of vesicles, thus complicating the purification of homogenous populations of pore particles suitable for crystallization trials.

Here we reveal structures of FraC corresponding to four different stages of its activation route, namely the water-soluble form, the lipid-bound form, an assembly intermediate and the transmembrane pore. Thermodynamic, functional and mutational data complement the structural analysis. Overall, we provide a detailed account of the activation of a α-PFT at the atomic level. These data clarify key aspects of the mechanism of action of actinoporins, and reveal critical roles for lipids in the activation and architecture of a PFT.

## Results

### Structure of the pore

**Figure 1:**
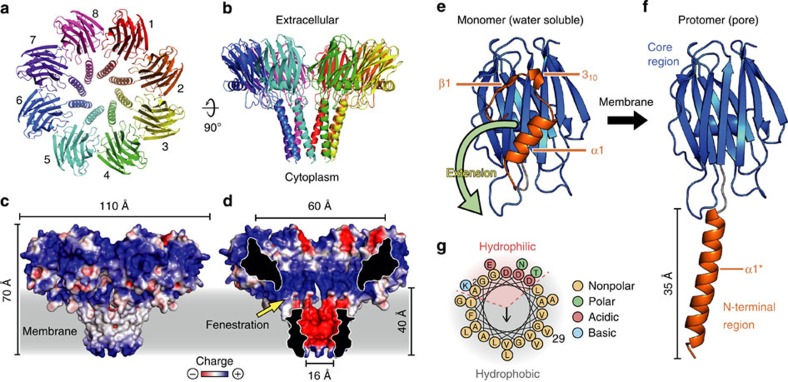
Structure of the transmembrane pore of FraC. (**a**) Top view. The pore is composed of eight identical protein chains (protomers). Lipid molecules are not shown. (**b**) Side-view showing the funnel-like shape of the pore. (**c**) Electrostatic potential of the outer surface of the pore. The colour gradient from red to blue represents negative (−3 kT) to positive electrostatic potentials (+3 kT). The plasma membrane region is represented in gray. (**d**) Electrostatic potential in the lumen of the pore. Colour scheme is same as above. Cross-section reveals distinctive fenestrations (windows) exposing the hydrophobic core of the membrane to the aqueous phase. (**e**) Representative structure of water-soluble (monomeric) FraC. The N-terminal region and the β-core are depicted in orange and blue, respectively. (**f**) Structure of a protomer of FraC inserted in the membrane. A large conformational change involving the first 29 residues of the N-terminal region (orange), but not the β-core, is clearly observed. (**g**) Helical wheel representation of the N-terminal amphipathic α-helix inserted in the membrane.

The crystal structure of the transmembrane pore of FraC was determined at 3.1 Å resolution (Fig. 1, Table 1, Supplementary Fig. 1). Diffraction quality crystals were obtained in lipidic mesophases^19^ using homogeneous pore particles prepared by first incubating water-soluble FraC with lipid vesicles, followed by two purification steps in the presence of detergents (Supplementary Fig. 2). The pore of FraC forms a symmetrical and funnel-shaped particle comprising eight identical protein chains (Fig. 1a,b). The pore particle has an outer diameter of 110 Å, and a height of 70 Å. These dimensions are consistent with cryo-EM reconstruction images of transmembrane pores of FraC inserted in liposomes^17^. The inner diameter of the pore ranges between 60 Å at the upper vestibule and 16 Å at its narrowest constriction near the cytoplasmic side. The lumen of the pore displays a negative electrostatic potential consistent with actinoporins being cation-selective channels (Fig. 1c,d)^12^.

Each protomer of FraC consists of a long transmembrane α-helix composed by residues of the N-terminal region (residues 4–29), and a core region rich in β-sheet structure that we termed β-core (residues 30–179). The secondary structure and conformation of the N-terminal region in the pore is profoundly altered with respect to that of the water-soluble monomeric protein (Fig. 1e,f). Some residues at the N-terminal region of the pore move more than 50 Å relative to their position in the water-soluble monomer (Supplementary Movie 1). The transmembrane α-helix spans the entire thickness of the membrane (>35 Å). The values of the hydrophobicity parameter (*H*=0.491) and hydrophobic moment (*μ*_H_=0.389) of this α-helix are consistent with its multimeric (that is, not monomeric) insertion in biological membranes (Fig. 1g)^20^. In contrast, the region corresponding to the β-rich region (β-core) remains essentially unchanged with respect to the water-soluble protein (root-mean-square deviation (rmsd)=0.4±0.1 Å). The metamorphosis of a relatively small percentage of residues during pore formation that we describe above is a common feature among β-PFT^8^,^10^,^21^. On the contrary, the only other α-PFT of know structure, ClyA, undergoes a much greater conformational change during the assembly of the pore involving ~50% of its entire sequence^7^.

It is interesting to note that the electrostatic potential seems to play an important role in the mechanism of action of FraC (Fig. 1c,d, Supplementary Fig. 3). The positive electrostatic potential on the membrane proximal region may enhance the initial adhesion of FraC to the surface of the membrane by establishing attractive interactions with the phosphate groups of the lipids. After the pore assembly has been completed, the negatively charged surface of the transmembrane helix acts as a cation-selective filter, as indicated above. However, it is unclear how the large hydrophobic surface exposed to the solvent is efficiently stabilized once the conformational change separating the N-terminal region from the β-core has occurred. Based on the data obtained with the PISA server^22^, the solvent-exposed surface of hydrophobic residues of the β-core of each protein chain increases by~400 Å^2^. The increase of solvent-exposed hydrophobic surface imposes a large energetic deficit that must be compensated with favourable energetic interactions somewhere else in the system, such as with attractive interactions between the protein and the membrane lipids (see below).

**Figure 2:**
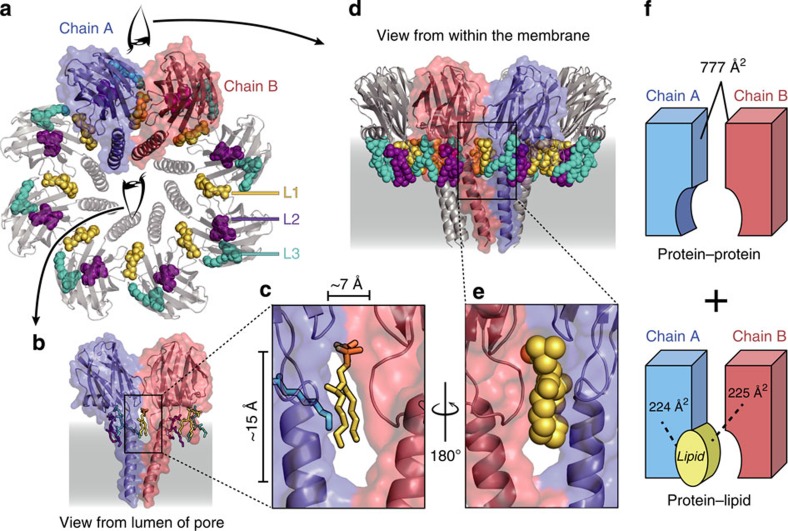
Lipids are integral part of the structure of the pore. (**a**) Top view of the pore depicting the bridging lipid (L1, yellow) and the annular lipids (L2, purple; L3, cyan). Lipids are depicted as space-filling models. (**b**) Lateral view (from the lumen of the pore) of two protein chains and the relative position of the associated lipids. The protein is shown as transparent surfaces and cartoons, and the lipids are depicted as solid sticks. (**c**) Close-up view of the non-annular lipid L1. (**d**) View of the pore from the lipid phase. (**e**) Close-up view of non-annular lipid L1 as seen from the lipid phase. (**f**) Schematic model of the composite interaction surface of the pore. The values correspond to the protein–protein contact interface and the lipid–protein contact interface as calculated by the PISA server^22^.

Intriguingly, the transmembrane pore displays eight lateral perforations (fenestrations), one at each protomer–protomer contact interface, partially occupied by the acyl-chain region of a bridging lipid (see below; Figs 1d and 2). The total area covered by these fenestrations is roughly 800 Å^2^ (~100 Å^2^ for each protomer–protomer interface). We note that this value is substantially greater than the area of the transmembrane pore at the narrowest constriction (~200 Å^2^).

Each pore contains 24 lipid molecules (all modelled as SM) firmly bound to the protein chains, that is, three lipids are associated to each protein chain (Fig. 2, Supplementary Fig. 4). We termed each lipid L1, L2 and L3. These tightly bound lipids are not exchanged with molecules of detergent during the solubilization of the proteoliposomes, the purification of the pore or the crystallization in the presence of high concentration of monoolein lipids (Supplementary Fig. 2). Notably, the lipid L1 is located between two adjacent protein chains partially covering the fenestrations described above (Fig. 2c,e,f). The lipids L2 and L3 are bound to single protein chains at regions rich in aromatic residues. All the lipids are located on the same plane, revealing the orientation of the pore with respect to the membrane (Supplementary Fig. 4). The lipid headgroup of these lipids engages in numerous non-covalent interactions with conserved residues of the protein, including H-bonds, and electrostatic and cation-π interactions, thus explaining their firm adhesion to FraC (Supplementary Fig. 4).

### Molecular basis for the stabilization of the pore

The architecture of the pore is held in place by intermolecular protein–protein (Supplementary Tables 1 and 2) and protein–lipid interactions (Supplementary Table 3). The protein–protein interaction surface comprises residues of the β-core (531±4.9 Å^2^) and residues of the transmembrane α-helix (246±6.9 Å^2^); Supplementary Table 1). The major contributors to the protein–protein interaction surface are residues Trp149 and Val60 each burying 115±6 Å^2^ and 96±1 Å^2^ of surface area, respectively. Protein–protein interactions are reinforced by seven hydrogen bonds between pairs of protomers (Supplementary Table 2). The average protein–protein contact interface^22^ between each pair of protein chains is 777±8 Å^2^, a rather small value compared with that reported for other toxins of similar size and known structure (2,100–2,800 Å^2^; Supplementary Table 4). The percentage of residues identical or strongly conserved at the protein–protein interface in the actinoporin family is 74%, a value smaller than that for the residues involved in lipid–protein interactions (81%), and short of the overall conservation for the entire alignment (77%; Supplementary Fig. 5, Supplementary Table 5).

Importantly, the architecture of the pore is built on extensive interactions between the protein and the L1 lipid (Fig. 2; Supplementary Fig. 4). The interaction surface between FraC and L1 is 449±12 Å^2^, thus comprising a large fraction (37%) of the total contact interface in the transmembrane pore (Fig. 2f). In addition, the headgroup region of the lipid establishes numerous non-covalent interactions with conserved residues of the binding pocket (Supplementary Fig. 4). Notably, the H-bonds observed between the L1 lipid (modelled as a SM-like lipid) and residue Arg31 of the protein are absent when the lipid modelled in the electron density is phosphatidylcholine (not shown), explaining the preference of actinoporins for SM^23^.

### Effect of the lipid composition

**Figure 3:**
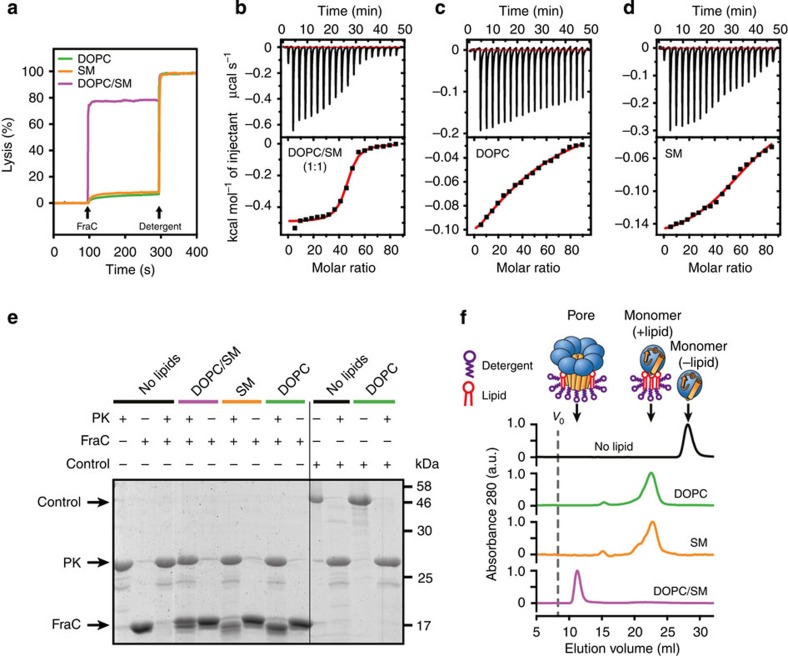
Functional analysis. (**a**) Lytic activity of FraC in vesicles made of different lipid compositions. Addition of protein and detergent are indicated by arrows. Complete lysis (100% signal) was obtained by adding detergent TX-100 at a final concentration of 0.1% v-v. (**b**–**d**) Characterization of binding of FraC to liposomes made of different lipid compositions by ITC. The upper panel corresponds to the titration. The bottom panel corresponds to the binding isotherm obtained from the titration curve. The red trace corresponds to the non-linear fitting of the integrated heat to a one-site model with ORIGIN^25^. The lipid composition is indicated in each panel (**b**, DOPC/SM (1:1); **c**, DOPC; **d**, SM). (**e**) Protection assay. FraC was treated with PK in the presence or absence of vesicles. A control experiment was performed with bovine serum albumin as a substrate, demonstrating that the activity of PK is not inhibited by liposomes. (**f**) Influence of the lipid composition in the assembly of FraC. Liposomes were treated with FraC, followed by solubilization with TX-100 and purified in the presence of LDAO. The black solid line corresponds to a control experiment in the absence of liposomes and detergents. The void volume (Vo) was 8 ml (vertical dashed line). FraC, detergent and lipid are represented using schematic cartoons. The orange rectangle and the blue sphere represent the N-terminal region and the β-core region of FraC, respectively.

To address the activation mechanism of FraC in membranes, we employed liposomes^24^ (Fig. 3). In actinoporins, pore formation is enhanced in the presence of SM and lipid-packing defects^15^,^16^, as shown in Fig. 3a. Liposomes made of one type of lipid (only SM or only 1,2-dioleoyl-*sn*-glycero-3-phosphocholine (DOPC)) were virtually insensitive to FraC (<10% lysis). In contrast, liposomes composed of a mixture of SM and DOPC (molar ratio 1:1) contain lipid domains that dramatically increase their susceptibility to the toxin (~80% lysis). The binding of FraC to liposomes is consistent with the lytic activity results, since the affinity is strongest in vesicles made of the equimolar mixture DOPC/SM (*K*_D_=0.22±0.04 μM) as determined by isothermal titration calorimetry (ITC; Fig. 3b–d). Binding of FraC to vesicles and its pore assembly are driven by a large change of enthalpy (Δ*H°*=−24.3±2.7 kcal mol^−1^) reflecting favourable non-covalent interactions, and opposed by a net loss of entropy (−*T*Δ*S*=15.3±2.8 kcal mol^−1^). This thermodynamic signature is consistent with that reported for sticholysin II (Stn-II, an actinoporin) titrated with vesicles made of an equimolar mixture of DOPC/SM/cholesterol^25^. Although the values of the binding constant of FraC to liposomes composed of SM (*K*_D_=5.8±0.7 μM) or DOPC (*K*_D_=48±6 μM) reveal lower affinity than that to vesicles of DMPC/SM, they nonetheless indicate a relevant adhesion to liposomes as demonstrated by the protection assay shown in Fig. 3e. In that experiment it is shown that the presence of vesicles, independent of its lipid composition, greatly protects FraC from the digestion with proteinase K (PK). The protection does not occur to a control protein that does not interact with liposomes. Consistent with the permeabilization assay described above, the formation of stable pores occurs only in vesicles of DOPC/SM as determined by size exclusion chromatography (SEC; Fig. 3f). Although the elution profile of the samples treated with vesicles of SM or with vesicles of DOPC is shifted to lower volumes with respect to the untreated sample, the oligomerization state of FraC does not increase, remaining in the monomeric state (Supplementary Fig. 2c). This unexpected observation raises the question of how the binding of lipids may affect the conformation of FraC.

### Crystal structure of the lipid-bound monomer

**Figure 4:**
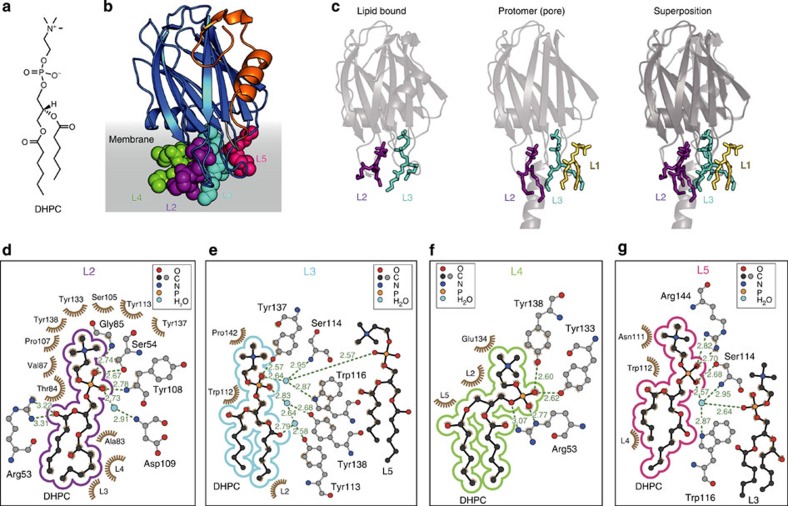
Structure of lipid-bound FraC. (**a**) Chemical formula of the lipid DHPC. (**b**) Overall structure of FraC with DHPC bound. The structure reveals up to four lipids bound to a single chain of FraC, although only two binding sites are conserved in all the protein chains refined (10 protein chains in three different spacegroups). The clustering of the lipids around FraC suggests the orientation of the protein with respect to the plane of the membrane. The lipids termed L2, L3, L4 and L5 are depicted as space-filling models and coloured in purple, cyan, green and magenta, respectively. (**c**) Depiction of the two constant lipid binding sites in the structure of FraC with bound DHPC (left panel), and in the pore (centre panel). The panel on the right shows the overlay of these two structures. The non-annular lipid L1 is absent in the DHPC-bound structure. (**d**–**g**) Environment around the four lipid molecules bound to an individual chain of FraC. The lipids shown are, from left to right, L2, L3, L4 and L5. Hydrogen-bond distances are shown in green.

To examine the lipid-bound conformation, we determined three independent crystal structures of FraC in complex with the water-soluble lipid 1,2-dihexanoyl-*sn*-glycero-3-phosphocholine (DHPC) at resolutions ranging from 1.6 to 2.3 Å (Fig. 4, Table 1, Supplementary Fig. 6). Remarkably, up to four lipid molecules are bound to a single protein chain. The molecules of DHPC bound to each molecule of FraC lie on a common plane, resembling the surface of a membrane. Although multiple molecules of DHPC bind to FraC, the toxin remains monomeric and its overall structure is virtually unchanged with respect to that of the water-soluble form (rmsd=0.37±0.10 Å). The examination of the structure in three different crystal forms reveals two well-conserved lipid-binding pockets, suggesting they are the primary sites during the initial adhesion of FraC to membranes (Supplementary Table 6). These two pockets overlap with those of lipids L2 and L3 seen in the structure of the transmembrane pore, and establish comparable non-covalent interactions with the lipids (Fig. 4c, Supplementary Fig. 6). In contrast, no evidence of the L1 lipid is found because the protein remains monomeric and, therefore, lacks a second protein chain contributing half of the binding site of L1. FraC–lipid interactions occur mainly at the headgroup region of the lipids. Because the headgroups of SM and DHPC are identical, that is, phosphorylcholine (POC), the crystal structures explain the targeting of actinoporins towards synthetic membranes containing these two types of lipids^24^.

Comparison of 10 independently refined protein chains (in three crystal forms) reveals two modest conformational changes in the protein. First, the loop comprising residues 77–85 at a region in contact with the membrane displays a broad range of movements with an amplitude of ~8 Å (Supplementary Fig. 6c). The flexibility of this loop may contribute to the anchoring of FraC to heterogeneous biological membranes displaying lipid defects. Second, the partial unfolding of residues 2–6 in one protein chain suggest a point of structural vulnerability facilitating the separation of the N-terminal region from the β-core, a necessary step for the activation of the transmembrane helix (Supplementary Fig. 6d).

### Crystal structure of an assembly intermediate

**Figure 5:**
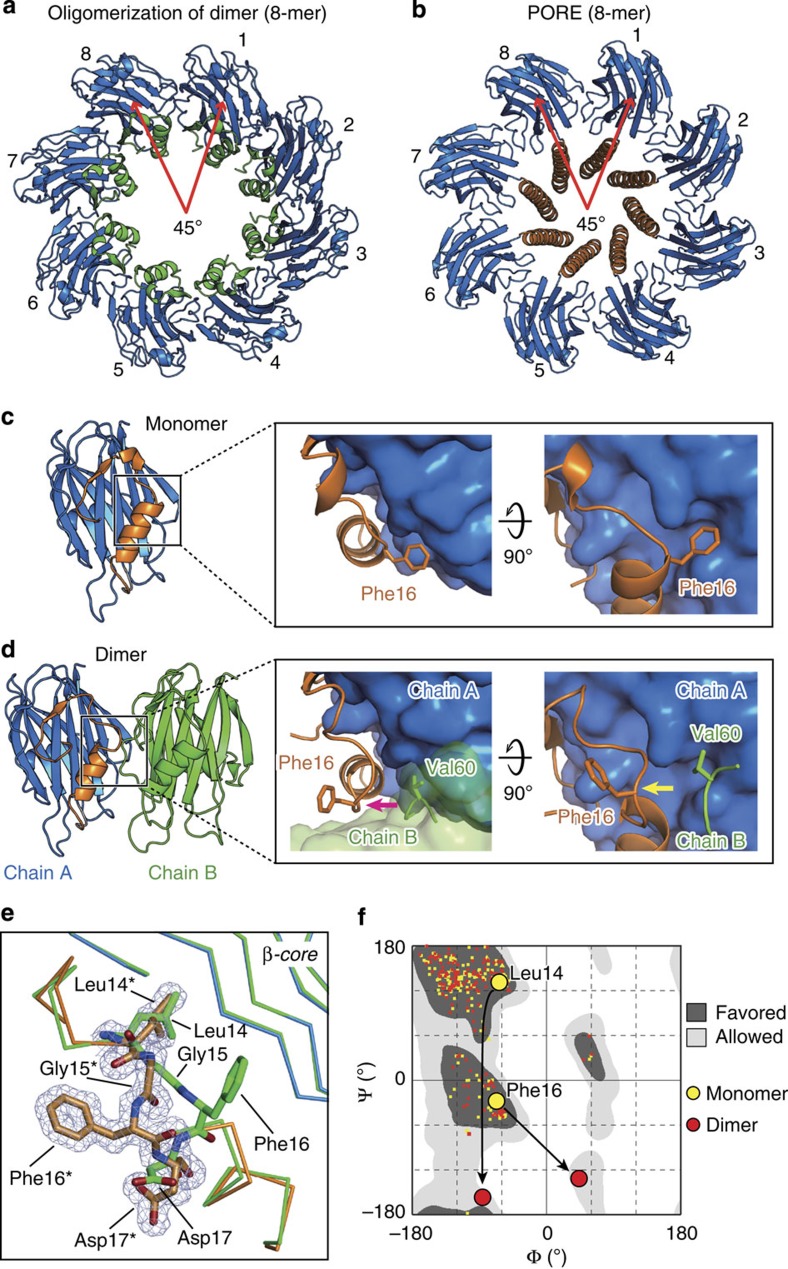
Structure of an assembly intermediate (dimer). (**a**) Top view of a hypothetical ‘pre-pore’ built from the assembly of successive dimers of FraC. To obtain this model, chain B of a fixed dimer and chain A of a mobile dimer were superimposed, after which chain A of the incoming chain was removed. This process was repeated until a complete rotation of 360 ° is obtained. Following this process yields a rotational 8-mer. We note that this octamer is not sealed at the ends. (**b**) Top view of the pore of FraC in the same orientation as above. (**c**) Crystal structure of water-soluble FraC (no lipids bound), and close-up view of the region where Phe16 interacts with the β-core region. (**d**) Crystal structure of dimeric FraC. Molecules of POC are not shown. The close-up view reveals conformational changes at Phe16 and adjacent residues with respect to the monomer. Dimerization is characterized by the displacement of the side chain of Phe16 by the residue Val60 of a second protein chain (green). (**e**) Close-up view of the conformational changes of residues 14–17 on dimerization. The mesh corresponds to the Sigma-A weighted electron density map (2Fo—Fc) contoured at *σ*=1.0. The chain corresponding to the monomer-like conformation is depicted in green. The main chain of the protein in the dimer-like conformation is shown in orange (N-terminal region) and blue (β-core). Residues labelled with an asterisk correspond to residues of the dimer-like conformation. (**f**) Ramachandran plot of water-soluble and dimeric FraC. The figure was prepared with RAMPAGE.

The crystal structure of a dimeric form of FraC in two crystal forms at 1.57 and 1.60 Å resolution sheds light on the initial events leading to the assembly of the pore (Fig. 5). These two high-resolution crystal structures are virtually identical to each other. Suitable crystals were obtained in the presence of POC (a compound that mimics the lipid headgroup of DOPC and SM) and the detergent dodecyl-β-maltoside (DDM). Two molecules of POC are bound to each protein chain, occupying identical positions to those of the headgroups of lipids L2 and L3 in the crystal structure of both, the transmembrane pore and the DHPC-bound monomer. The DDM moiety is not observed in the electron density maps. In contrast to FraC, the crystal structure of Stn-II displays only one molecule of POC bound to the monomeric protein^18^.

The protein–protein dimerization surface and the non-covalent interactions observed in the dimer resemble those between residues of the β-core region in the transmembrane pore (rmsd=0.4±0.04 Å; Supplementary Tables 1 and 2). Using the dimerization surface as a template, we built an oligomeric model composed of eight molecules very similar to the pore particle in terms of overall size and chain organization (Fig. 5a,b). This comparison reinforces the validity of the dimer as an assembly intermediate.

The structure of the dimer reveals a localized and notable conformational change with respect to the monomer (water soluble) at residues 14–17 of the N-terminal region. In the monomeric form of FraC, the side chain of Phe16 is inserted in a hydrophobic cavity of the β-core region (Fig. 5c). In contrast, during dimerization the residue Val60 of a second protein chain displaces Phe16 from its original position, leading to (i) partial unfolding and increase strain in the peptide bond of Phe16 and its neighbouring residue Leu14, (ii) a displacement of 4.7±0.1 Å of the Cα of Phe16 towards the solvent and (iii) further structural adjustments of Gly15 and Asp17 (Fig. 5d–f). Because the N-terminal region is glued to the β-core region, partly by interactions between apolar residues (Supplementary Fig. 3), the rearrangement of two large hydrophobic residues (Leu14 and Phe16) may facilitate the detachment of the N-terminal region towards the membrane, a necessary condition to generate the active pore.

These conformational changes were not observed in a previous structure of a non-lytic oligomeric form of FraC (9-mer) obtained in detergent LDAO (PDB entry code 3LIM)^17^. We argue that the absence of conformational changes in the 9-mer reflects the different angle between adjacent protein–protein chains (Supplementary Fig. 7). The relative rotation between adjacent protomers in the 9-mer form is 40°, whereas packing in the dimer (and pore) is more compact (45°) thus facilitating the conformational changes. The lower degree of protein–protein packing in the 9-mer results in fewer number of H-bonds and a smaller interaction surface (four H-bonds, buried surface area (BSA)=1,188±43 Å^2^) compared with those of the pore (seven H-bonds, BSA=1,554±18 Å^2^) or those of the dimer (six H-bonds, BSA=1,518±83 Å^2^; Supplementary Tables 1 and 2). The localized unfolding of the assembly intermediate (dimer) of FraC is thus more akin to the rearrangements observed in the pre-pore state of β-PFTs such as perfringolysin O^26^ or aerolysin^27^, than that in its own non-lytic 9-mer form^17^.

### Mutational analysis

Three different pairs of muteins modifying critical regions of FraC, namely the lipid binding site (W112R/W116F)^28^, the N-terminal region (V8C/K69C^OX^)^29^ and the protein–protein contact interface (V60E/W149A), demonstrate the essential role of these structural elements in the haemolytic activity of FraC (Supplementary Fig. 8). The haemolytic potency of the muteins decreases in the order V60E/W149A>V8C/K69C^OX^>>W112R/W116F, suggesting a hierarchy in the events conducive to the formation of the transmembrane pore. First, mutein W112R/W116F lacks the ability to bind to the membrane, thus becoming completely inactive. Second, blocking the extension and insertion of the N-terminal region in the membrane by engineering a disulfide-bond between the N-terminal and β-core regions (V8C/K69C^OX^) inactivates the toxin, in agreement with a previous report^29^. The low haemolytic activity (1% that of wild-type FraC), the high retention volume determined by SEC (22.5 ml) and the different thermodynamic binding profile indicates that this mutein does not form stable pores in susceptible DOPC/SM membranes. Third, the mutein V60E/W149A designed to weaken the stability of the pore elutes as a monomeric protein by SEC (22.3 ml) and displays a thermodynamic profile similar to that of V8C/K69C^OX^. However, the haemolytic activity of V60E/W149A is closer to that of wild-type FraC (~16% that of wild type), suggesting that the mutein V60E/W149 forms functional but otherwise structurally weak pores.

## Discussion

**Figure 6:**
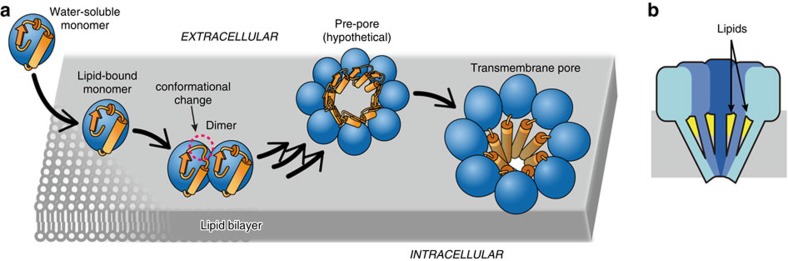
Model for pore formation by FraC in biological membranes. (**a**) Stepwise mechanism. Water-soluble monomeric FraC binds to multiple lipids through the POC moiety of their headgroups. On binding, protein–protein interactions on the plane of the membrane facilitate dimerization and partial unfolding at the N-terminal region (red circle). In the presence of lipid rafts, coalescence of multiple chains of FraC leads to further unfolding, triggering the insertion of the N-terminal region inside the target membrane and the formation of the active transmembrane pore. The N-terminal region and the β-core region of FraC are schematically represented by orange rectangles and blue spheres, respectively. (**b**) Model of the hybrid protein/lipid pore of FraC. The arrows indicate the structural lipids (yellow) lining the wall of the pore and acting as assembly cofactors.

In this study we have revealed the structural basis for the spontaneous metamorphosis of a water-soluble protein into a transmembrane haemolytic pore. The structural snapshots demonstrate that the assembly of the pore can be described as a stepwise process (Fig. 6a). Importantly, the pore is made not only of protein molecules, but also of lipid molecules (Fig. 2, Supplementary Fig. 4). In particular, we identified lipid molecules lining the wall of the pore, as schematically represented in Figs 2f and 6b. The identity of this lipid is SM, which not only acts a receptor for FraC on the surface of the membrane^14^,^23^, but also as a *bona fide* structural element playing the role of an assembly co-factor. The presence of this lipid seems to be a unique feature of actinoporins, since it has not been observed in the crystal structures of the transmembrane pores of β-PFTs^8^,^9^ or of ClyA^7^ (an α-PFT). The presence of a structural lipid lining a transmembrane pore is thus unprecedented among PFTs, and resembles the essential non-annular lipids bound to integral membrane proteins^30^. The key structural role of the non-annular L1 lipid suggests an sophisticated mechanism by which FraC identifies the target membrane, since the presence of this lipid in the susceptible membranes will also govern the assembly of the toxin.

In addition to the critical role of SM in the assembly of the final pore, the model proposed in Fig. 6a advances our understanding of actinoporins, and that of PFT in general, in three additional aspects: (i) the presence of multiple lipid binding sites in a PFT (lipid multivalency), (ii) the architecture of an assembly intermediate (dimer) and (iii) the presence of fenestrations on the wall of the transmembrane pore.

First, our results demonstrate the concept of lipid multivalency in a PFT (Fig. 4), a property of FraC that may increase the affinity of the toxin for the membrane during the early stages of membrane binding. The observation of up to four lipid binding sites in FraC advances the model by Mancheño *et al*.^18^, in which it was proposed that the actinoporin Stn-II possesses a single lipid binding site (identified with POC as a probe; Fig. 4). Judged from the occupancy of the lipid molecules bound to FraC, we propose two high-affinity sites (corresponding to lipids L2 and L3). These two binding pockets are suited to recognize the solvent-exposed region of the lipids, that is, the POC headgroup. In contrast, the pockets engaging lipids L4 and L5 represent low-affinity sites, or perhaps high-affinity binding sites for lipids with headgroups other than POC.

Second, the serendipitous capture of a dimeric form of FraC exhibiting a similar protein–protein interaction interface to that of the pore, suggests a mechanism initiating the self-assembly and metamorphosis of FraC. The high-resolution data (1.6 Å resolution) reveal localized conformational changes at the dimer interface (mainly residues Leu14−Phe16) with respect to the monomeric protein (Fig. 5). Together with the unfolding of the N terminus (Supplementary Fig. 6) these data suggest the existence of ‘weak’ spots from which the extension of the N-terminal region towards the membrane originates. The observation of a stable assembly intermediate contrasts with the mechanism proposed for β-PFTs, in which no lower order intermediates are detected^31^.

Interestingly, the crystal structure of a non-lytic 9-mer ‘pre-pore’ species obtained in the presence of the detergent LDAO has been described^17^,^32^. In those two studies, high-order transmembrane pores embedded in liposomes made of DOPC/SM are also reported, based on the reconstruction of symmetry-averaged cryo-EM images at ~30 Å resolution. Although no conclusive proof of a transmembrane 9-mer versus an 8-mer pore can be drawn at that resolution, the possibility that FraC forms active pores with more than one stoichiometry is an attractive idea and a well-documented feature for other PFTs. Transmembrane pores of variable stoichiometry have been reported for ClyA (12- and 13-mer)^7^,^33^, the protective antigen of the anthrax toxin (7- and 8-mer)^34^, and the large pneumolysin pore (38- to 44-mer)^35^. However, based on the evidence presented here, we propose that the 8-mer is the predominant transmembrane pore of FraC in DOPC/SM vesicles because (i) only the 8-mer species is observed in the crystal structure of the transmembrane pore, and (ii) the protein–protein packing interface in the extracellular region of the 8-mer is more favourable than that of the 9-mer (Supplementary Fig. 7, Supplementary Tables 1 and 2). Since the lipid plays an important role in pore formation, it will be interesting to determine if the composition of the acyl-chain of SM or the identity of the headgroup of the lipid governs the stoichiometry of the pore.

Third, the aqueous channel exhibits fenestrations (windows) in the walls of the pore (Figs 1 and 2). Such fenestrations have not been previously documented in PFTs or viroporins^36^,^37^. Similar perforations have been recently described in two different classes of ion channels, although their exact biological role remains unknown^38^,^39^. These fenestrations are partially occupied by the hydrophobic region of the lipid L1, although the dynamic nature of its acyl chains (suggested from the poor electron density features) prevents the formation of a rigid plug sealing off the solvent (Fig. 2, Supplementary Fig. 4). Because of the imperfect features of this seal, we suggest that the fenestrations could facilitate the diffusion of small hydrophobic molecules present in the venom of sea anemones^40^ directly inside the core of the membrane, thus intensifying cell damage. The lateral section of small molecules fitting through the unobstructed fenestrations is ~5 × 11 Å (based on the rigid crystal structure and considering the van der Waals radii). The hypothetical diffusion of these small molecules in the membrane through the fenestrations could be energetically advantageous in comparison with the entrance through the more compact water-membrane interface on the surface of the cell. In addition, the amphipathic character of the fenestrations may contribute to local disruption of the lipidic lamellar structure, for example, catalyzing the transbilayer (flip-flop) movement of lipids.^41^,^42^

In summary, we have revealed the structural basis for the spontaneous metamorphosis of a water-soluble protein into a haemolytic transmembrane pore lined by protein and lipid. The presence of specific lipids in the structure of the pore ensures that the haemolytic pore is assembled only in membranes containing the lipids that activate the protein. We anticipate these concepts will deepen our general understanding of membrane proteins and PFT, and will be of applicability for the design of nanopores and the engineering of drug delivery systems.

## Methods

### Materials

SM from porcine brain, DOPC and DHPC were purchased from Avanti Polar Lipids (Alabaster, AL, USA). The soluble lipid dehydroepiandrosterone was obtained from TCI (Tokyo, Japan). The disodium salt of 8-aminonaphthalene-1,3,6-trisulfonic acid (ANTS) and p-xylene-bis-pyridinium bromide (DPX) were purchased from Molecular Probes (Eugene, OR, USA). Bovine serum albumin and detergent LDAO were obtained from Sigma-Aldrich (St Louis, MO, USA). DDM was obtained from Dojindo (Tokyo, Japan). Triton X-100 was purchased from Anatrace (Maumee, OH, USA). Wide-range molecular weight marker was from Bio-Rad (Hercules, CA, USA). PK was purchased from Boehringer Ingelheim (Ingelheim, Germany). Reagents for the crystallization of proteins were obtained from Hampton Research (Aliso Viejo, CA, USA) or Molecular Dimensions (Newmarket, UK). Other chemicals were from Wako (Tokyo, Japan).

### Preparation of FraC and muteins

FraC were expressed and purified as described previously^11^ with some modifications. Briefly, *E. coli* BL21 (DE3) cells were transformed with a vector containing the sequence of FraC, and grown at 37 °C. Expression was induced with 1 mM isopropyl β-D-1-thiogalactopyranoside for 5 h, and cells subsequently harvested by centrifugation (8,000*g*, 10 min, 4 °C). The cell-pellet was resuspended in buffer A (50 mM Tris at pH 7.4), and subsequently lysed with an EmulsiFlex C-5 homogenizer (Avestin, Canada). The lysate was centrifuged at 40,000*g* for 30 min at 4 °C. The supernatant was filtered with a Millex GP 0.22 μm unit (Merck Millipore, Darmstadt, Germany), and then applied to a Resource S cationic-exchange column (GE Healthcare, Piscataway, NJ, USA) equilibrated with buffer A. FraC was eluted with buffer B (50 mM Tris, and 1 M NaCl, pH 7.4), concentrated and further purified by SEC in a HiLoad 16/60 Superdex 75 pg column (GE Healthcare) equilibrated with SEC buffer (50 mM Tris, 200 mM NaCl, pH 7.4). Purity was at least 98% homogeneous as judged by SDS–PAGE (not shown). All FraC mutations were prepared by site-directed mutagenesis using KOD-Plus Mutagenesis Kit (Toyobo, Japan).

### Preparation of liposomes

The appropriate amount of lipids dissolved in chloroform were mixed and thoroughly dried in an evaporator, hydrated with SEC buffer and subjected to 10 cycles of freeze thaw. Liposomes were prepared by the extrusion method using polycarbonate filters with a pore diameter of 100 nm (Nucleopore, Pleasanton, CA, USA) in a Mini-Extruder apparatus (Avanti). Lipid concentration was determined by the method of Bartlett^43^.

### Purification of oligomeric FraC

Monomeric FraC (50 μM) was incubated with LUVs (lipid/protein molar ratio was 200:1) in SEC buffer for 30 min at room temperature, followed by solubilization of the liposomes with Triton X-100 at 1% v-v (Supplementary Fig. 2). The solution was applied to a Resource S column equilibrated with buffer A and LDAO (3 mM) to remove the solubilized lipids and the excess Triton X-100 from the protein. FraC was eluted with buffer B supplemented with 3 mM LDAO, and subsequently analyzed by analytical SEC in a Superdex 200 10/300 GL column equilibrated with SEC buffer and 3 mM LDAO.

### Crystallization of the water-soluble FraC

Systematic and microseeding crystallization screens were carried out in an Oryx8 instrument (Douglas Instruments, UK). Irregular-shaped crystals of FraC were obtained by mixing 2 μl of fresh purified protein at 10 mg ml^−1^ in 10 mM Tris HCl (pH 8.0) and 2 μl of crystallization solution composed of 16% PEG 8,000, 200 mM (NH_4_)_2_SO_4_ and 100 mM sodium cacodylate (pH 7.4). These crystals were processed as described previously to obtain a solution of stock seeds^44^,^45^. Single crystals suitable for X-ray diffraction analysis were obtained by mixing 2 μl of fresh purified protein at 10 mg ml^−1^ with 1.8 μl of crystallization solution composed of 20% Jeffamine and 100 mM HEPES (pH 7.4) and 0.2 μl of stock seeds. Rod-like crystals grew to a final size of ~0.6 × 0.2 × 0.2 mm^3^ after 2–3 weeks at 20 °C. Suitable crystals were identified, harvested, immersed in a solution of mother liquor supplemented with 20% glycerol and subsequently frozen by immersion in liquid nitrogen. A second type of crystal of FraC (in space group P2_1_2_1_2_1_) was obtained by mixing 2 μl of fresh purified protein at 10 mg ml^−1^ in 10 mM Tris/HCl (pH 8.0) and 2 μl of crystallization solution composed of 18% PEG 4,000, 20% (v/v) 2-propanol and 100 mM sodium citrate (pH 5.6). Crystals were frozen as outlined above, except that the cryoprotective solution contained mother liquor supplemented with 25% GalNAc. Both types of crystals were stored in liquid N_2_ until data collection.

### Crystallization of lipid-bound FraC

Crystals of FraC bound to the lipid DHPC were obtained by the co-crystallization method in three different space groups using the hanging-drop vapour difussion technique. Crystals in spacegroup P3_2_ were obtained by mixing equal volumes of FraC and a solution containing 0.2 M sodium thiocyanate, 20% PEG 3,350, 30 mM DHPC and 3 mM dehydroepiandrosterone (pH 6.9). Crystals in spacegroup P4_2_2_1_2 and P321were obtained by mixing equal volumes of FraC and a solution containing 50 mM DHPC, 200 mM Li_2_SO_4_, 20% PEG 1,000 and 100 mM phosphate-citrate buffer at pH 4.2. Crystals grew within a few weeks at 20 °C. Cryoprotection of crystals in spacegroup P321 was obtained by supplementing the crystallization solution with 20% glycerol. Crystals in spacegroup P4_2_2_1_2, were frozen after a 2-day dehydration treatment in the same crystallization solution supplemented with 35% PEG 1,000 and stored in liquid N_2_ until data collection.

### Crystallization of POC-bound FraC

Crystals of FraC with bound POC were obtained in two different spacegroups. Crystals in spacegroup P4_3_2_1_2 were obtained by mixing equal volumes of FraC and a solution containing 100 mM POC, 3 mM DDM, 200 mM Li_2_SO_4_, 100 mM ammonium formate, 24% PEG 8,000 in a buffer of 100 sodium acetate in the pH range of 4.1−4.3. Crystals grew within a few weeks at 20 °C. Crystals in spacegroup P2_1_ were obtained under the same conditions except that ammonium formate was absent from the crystallization solution. Cryoprotection was achieved by supplementing the mother liquor with 20% glycerol.

### Crystallization of the transmembrane pore

Crystals of the transmembrane pore were obtained from solubilized oligomers of FraC using the procedure outlined in Supplementary Fig. 2. For the crystallization experiments, we employed DDM 0.015% (w:v) instead of LDAO. Crystals were obtained by mixing 1 μl of FraC at 7 mg ml^−1^ in 20 mM Tris/HCl (pH7.4), 10 mM NaCl and 0.015% DDM, and a lipidic solution containing Jeffamine M600, 1,2,3-heptanetriol, monoolein, ammonium sulfate and HEPES at pH 7.0 using the lipid sponge phase method^19^. The reservoir contained 0.1 g l^−1^ Na/KPO_4_, 0.55 M sodium acetate and 0.75 M HEPES (pH 6.2). Crystals developed to full size in ~6 months at 20 °C. Suitable crystals were harvested and frozen by direct immersion in liquid N_2_.

### Data collection and refinement

Data collection was carried out at beamlines BL-5A, AR-NE3A and AR-NW12 of the Photon Factory (Tsukuba, Japan) under cryogenic conditions (100 K). The structure of the water-soluble form was determined by the molecular replacement method using the coordinates of FraC (PDB entry code 3LIM) with PHASER^46^. For the other crystal structures, the coordinates of the water-soluble monomer were used as the initial model for the molecular replacement step. The coordinates were refined with REFMAC5 (ref. 47) and COOT^48^ from the CCP4 suite. The quality of the final model was assessed with COOT and PROCHECK^49^, as well as with the validation tools provided by the Protein Data Bank^50^. The coordinates and topology file of DHPC and SM were generated with PRODRG^51^. Data collection and refinement statistics are shown in Table 1. We note that the structure of the transmembrane pore was modelled with 17 water molecules (2.4% of the total number of residues in the asymmetric unit). Of the 17 water molecules modelled, 15 are conserved in the higher resolution structures of the water-soluble monomer or the lipid-bound form. The additional two water molecules engage the crucial L1 lipid, and together with the L1 lipid itself, are not observed in the high-resolution structures.

### Leakage of liposome contents

The leakage of encapsulated solutes was assayed by the method of ANTS/DPX^52^. LUVs containing ANTS and DPX were prepared as described previously^53^. Changes in fluorescence intensity were recorded in a FluoroMax-3 spectrofluorometer (Horiba, Japan) with excitation and emission wavelengths set at 355 nm and 515 nm, respectively. A 475 nm filter was placed between the sample and the emission monochromator. Liposome concentration was 100 μM. Toxin was added at a final concentration of 0.6 μM (lipid/protein molar ratio was 150:1). The complete release of the fluorescent probe (100% signal) was achieved by the addition of Triton X-100 (final concentration=0.1% w/v). Measurements were carried out at 25 °C with constant stirring. The levels of encapsulation were similar in all the compositions of LUV tested (not shown).

### Protease protection assay

PK (1.78 μg, 6 μM) was incubated with FraC (4.8 μg, 24 μM) for 24 h (FraC/protease molar ratio=4:1) in 50 mM Tris, 200 mM NaCl, 1 mM CaCl_2_ at pH 7.8. In the assays with lipids, FraC was incubated with the appropriate liposomes (3.65 mM lipid) for 30 min prior to the addition of PK into a final volume of 10 μl. The reaction was stopped by adding 2 μl of PMSF (final concentration=5 mM). Bovine serum albumin (4.8 μg, 7.2 μM) was used as a control.

### Haemolytic assay

Defibrinated horse blood (Nippon Biotest Laboratories, Japan) was washed with buffer (20 mM HEPES, 150 mM NaCl, pH 7.5) until the supernatant was clear. Haemolytic activity was determined by twofold serial dilutions of FraC in a 96-well titration plate with buffer in a total volume of 200 μl. After an incubation period of 60 min at room temperature, samples were centrifuged, and 100 μl of supernatant was diluted with 1 ml of buffer. The absorbance of haemoglobin released was measured in a V-660 Spectrophotometer (Jasco, Japan) at a wavelength of 400 nm. Alternatively, the time course of haemolysis was monitored at 600 nm and a protein concentration of 20 nM.

### Isothermal titration calorimetry

Calorimetric data was adquired in an ITC200 Micro Calorimeter (GE Healthcare) in SEC buffer at 25 °C. The LUV suspension at a lipid concentration of 10–20 mM was injected into the cell containing FraC at 20–51 μM under constant stirring (1,000 r.p.m.). The binding isotherms were fitted to a model in which a molecule of protein binds to ‘*n*’ molecules of lipid, as described previously^25^.

### SEC with multi-angle light scattering detection (SEC-MALS)

The elution peak of FraC incubated with DOPC was analyzed by analytical SEC in a WTC-030S5 column installed in a Heleos 8+ instrument (Wyatt Technology, Santa Barbara, CA, USA) equipped with a triple MALS/refraction index (RI)/ultraviolet detector. The total mass (21.1±1.9 kDa; red), the mass of FraC (20.0±1.8 kDa; blue) and the detergent LDAO (1.1±1.8 kDa; green) were calculated from MALS/RI/UV output. For FraC, the values of d*n*/d*c* and *ε* at 280 nm were 0.185 ml g^−1^ and 2,221 ml g^−1^ cm^−1^, respectively^54^. For LDAO, the values of *d*n/*d*c and *ε* at 280 nm were 0.148 ml g^−1^ and 0.003 ml g^−1^ cm^−1^, respectively. The elution peak of FraC incubated with DOPC/SM and solubilized with DDM was analyzed in the same manner as the monomer of FraC. The total mass (395±4 kDa; red), the mass of FraC (167±2 kDa; blue) and that of DDM (228±5 kDa; green) were calculated from the MALS/RI/UV signal. For DDM, the values of d*n*/d*c* and *ε* at 280 nm were 0.133 ml g^−1^ and 0.003 ml g^−1^ cm^−1^, respectively^54^.

## Author contributions

All authors designed the research. K.T., J.M.M.C. and K.M. performed the experiments. All authors analyzed the data. K.T. and J.M.M.C. wrote the paper with input from the all other authors.

## Additional information

**Accession codes.** Atomic coordinates and structure factors for the crystal structure have been deposited in the Protein Data Bank (PDB) under accession codes 3W9P and 3VWI for water-soluble monomer; 4TSP, 4TSQ, and 4TSO for the DHPC-bound monomer; 4TSL and 4TSN for the dimer; and 4TSY for the transmembrane pore.

**How to cite this article:** Tanaka, K. *et al*. Structural basis for self-assembly of a cytolytic pore lined by protein and lipid. *Nat. Commun.* 6:6337 doi: 10.1038/ncomms7337 (2015).

## Supplementary Material

Supplementary Information Supplementary Figures 1-8, Supplementary Tables 1-6 and Supplementary References

Supplementary Movie 1 Animation of the conformational change of a molecule of FraC from the monomer water-soluble state (orange) to the transmembrane pore state (blue). The animation was prepared with the program CHIMERA

## Figures and Tables

**Table 1:** Data collection and refinement statistics*.

	**Water soluble (I)**	**Water soluble (II)**	**Dimer, POC (I)**	**Dimer, POC (II)**	**Lipid bound (I)**	**Lipid bound (II)**	**Lipid bound (III)**	**Pore**
*Data collection*								
Space group	P 1 2_1_ 1	P 2_1_ 2_1_ 2_1_	P 4_3_ 2_1_ 2	P 1 2_1_ 1	P 3 2 1	P 4_2_ 2_1_ 2	P 3_2_	C 2 2 2_1_
								
*Cell dimensions*								
*a*, *b*, *c* (Å)	77.2, 44.3, 114.7	59.0, 60.5, 80.8	64.7, 64.7, 219.8	67.1, 65.6, 86.7	131.1, 131.1, 49.5	129.6, 129.6, 49.2	70.3, 70.3, 203.0	151.3, 199.9, 120.6
*α*, *β*, *γ* (°)	90.0, 92.8, 90.0	90.0, 90.0, 90.0	90.0, 90.0, 90.0	90.0, 101.1, 90.0	90.0, 90.0, 120.0	90.0, 90.0, 90.0	90.0, 90.0, 120.0	90.0, 90.0, 90.0
Resolution (Å)	37.1–1.70 (1.79–1.70)	42.2–2.10 (2.21–2.10)	32.4–1.60 (1.67–1.60)	35.7–1.57 (1.65–1.57)	32.8–2.30 (2.42–2.30)	38.9–2.15 (2.27–2.15)	45.3–1.60 (1.69–1.60)	38.7–3.14 (3.32–3.14)
*R*_merge_†	0.068 (0.291)	0.104 (0.366)	0.103 (0.801)	0.090 (0.480)	0.069 (0.496)	0.125 (0.567)	0.045 (0.136)	0.153 (0.411)
*I*/*σI*	12.8 (3.3)	13.5 (4.1)	17.5 (3.9)	9.6 (2.3)	18.8 (3.3)	9.5 (2.0)	17.0 (5.0)	9.9 (3.4)
Completeness (%)	93.7 (75.1)	100 (100)	100 (100)	98.4 (96.0)	97.5 (95.7)	93.5 (93.8)	95.4 (77.1)	94.6 (83.5)
Multiplicity	3.5 (2.9)	6.4 (4.8)	13.8 (14.1)	3.6 (3.1)	8.4 (6.1)	3.1 (3.0)	3.3 (2.0)	7.3 (5.5)
								
*Refinement*								
Resolution (Å)	37.1–1.70 (1.74–1.70)	42.2–2.10 (2.15–2.10)	32.4–1.60 (1.64–1.60)	35.7–1.57 (1.61–1.57)	32.8–2.30 (2.36–2.30)	38.9–2.15 (2.21–2.15)	45.3–1.60 (1.64–1.60)	38.7–3.14 (3.32–3.14)
No. reflections (test)	80,105 (3,182)	17,450 (883)	62,832 (1,910)	101,755 (4,087)	21,323 (1,106)	22,672 (1,167)	141,075 (4,325)	30,474 (1,526)
*R*_work_/*R*_free_ (%)‡	15.3/19.4 (20.2/27.2)	16.2/21.6 (18.8/23.0)	14.9/17.2 (24.1/26.8)	16.0/19.7 (24.2/27.1)	18.2/22.7 (28.0/33.9)	20.0/24.7 (26.4/31.7)	13.2/15.5 (18.8/21.2)	19.9/22.1 (27.2/34.8)
								
*No. atoms*								
Protein	5691	2746	2914	5740	2784	2789	8545	5504
Ligand	—	—	77	88	150	120	598	356
Other (not water)	32	—	50	74	45	61	17	105
Water	729	148	361	718	116	116	877	17
								
*B-factors (Å*^*2*^)								
Protein	18.1	20.5	18	18	43.5	35.9	19.7	52.4
Ligand	—	—	21.1	16.7	64.7	49.1	40.1	68.1
Other (not water)	30.9	—	32.1	41.3	83.2	57.3	23.5	83.9
Water	28.7	22.8	29.1	28.5	40.5	28.6	24.3	25.5
								
*R.m.s deviations*								
Bond lengths (Å)	0.020	0.011	0.022	0.017	0.007	0.012	0.019	0.013
Bond angles (°)	1.95	1.44	2.33	1.87	1.57	1.98	2.2	1.7
PDB ID	3VWI	3W9P	4TSL	4TSN	4TSO	4TSP	4TSQ	4TSY

POC, phosphorylcholine; R.m.s., root mean square.

^*^Highest resolution shell is shown in parenthesis.

^†^*R*_merge_=ΣhklΣ*i* |*I*(hkl)*i*—[*I*(hkl)]|/Σhkl Σ*i I*(hkl).

^‡^*R*_work_*=*Σhkl|*F*(hkl)_o_—[*F*(hkl)_c_]|/Σhkl *F*(hkl)_o_; *R*_free_ was calculated as *R*_work_, where *F*(hkl)_o_ values were taken from 3–5% of data not included in the refinement.
