# Supplementary material for: Structural basis for self-assembly of a cytolytic pore lined by protein and lipid
Source: Nat Commun. 2015 Feb 26;6:6337. doi: 10.1038/ncomms7337 (PMC4351601; doi:10.1038/ncomms7337)
Supplement: Supplementary Information — Supplementary Figures 1-8, Supplementary Tables 1-6 and Supplementary References [file ncomms7337-s1.pdf]

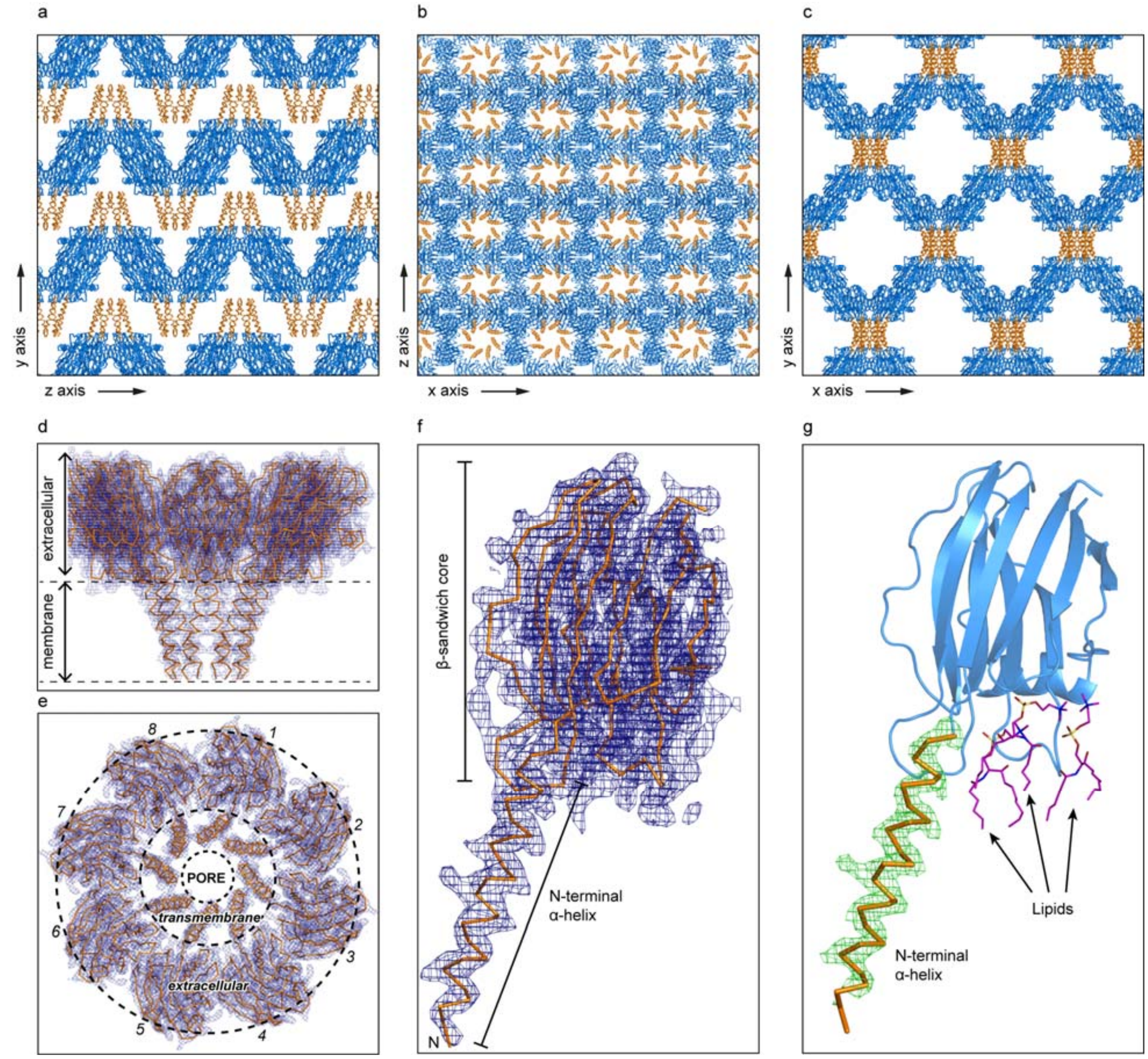

Supplementary Figure 1 | Overall structure of the transmembrane pore of FraC. a, Crystal packing of the pore along the y-z, **b,** the z-x, and **c,** the y-x planes. The N-terminal and the β-core regions are depicted in blue and orange, respectively. We note panels are not set in the same scale. **d,** Side-view. Sigma-A weighted 2Fo − Fc electron density map contoured at σ = 1.0 corresponding to the complete pore particle (the complete pore comprises two identical asymmetric units, each formed by four protein chains). **e,** Top view. **f,** Sigma-A weighted 2Fo − Fc electron density map of a protomer. The long N-terminal α-helix protrudes from the β-core region. **g,** Sigma-A weighted difference *omit* electron density map (Fo − Fc) of the transmembrane α-helical region at a contouring level of σ = 3.0. The electron density map (green) was calculated *before* the α-helix was modeled in the structure. Lipids are shown as sticks.

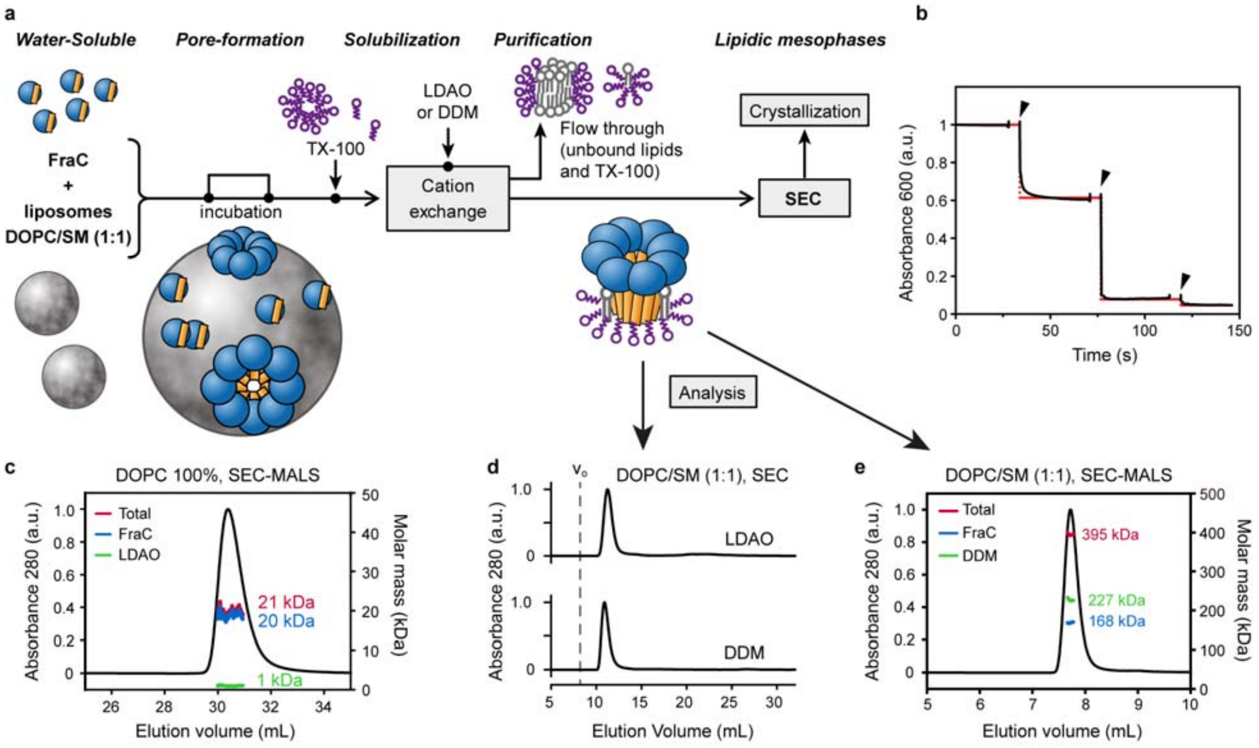

Supplementary Figure 2 | Assembly and purification of the pore. **a,** Procedure to assemble the pore using lipid vesicles, followed by solubilization with detergents. The protein-detergent complexes were purified by ion-exchange chromatography and SEC and, subsequently, used for crystallization trials using the lipid-sponge method[1]. **b,** Representative solubilization profile of vesicles composed of SM/DOPC (1:1) using the detergent Triton X-100. The concentration of lipid was 10 mM. Turbidity was monitored in a Jasco V-660 spectrophotometer at a wavelength of 600 nm in a thermostated cell holder at 25 °C. The arrows indicate successive addition of 10 µL-aliquots of Triton X-100 (10% v:v) to a solution containing 600 µL of vesicles. **c,** Molecular weight of the lipid-bound monomer of FraC in the presence of detergents was determined by SEC-MALS (MW = 20.0 ± 1.8 kDa; blue). The black trace corresponds to the UV signal at 280 nm. **d,** Purification of the pore of FraC using the detergent LDAO (top) or DDM (bottom). No significant differences are observed. **e,** Molecular weight of the pore of FraC determined by SEC-MALS[2]. The methodology is described in detail in the methods section (MW = 168 ± 2 kDa; blue trace).

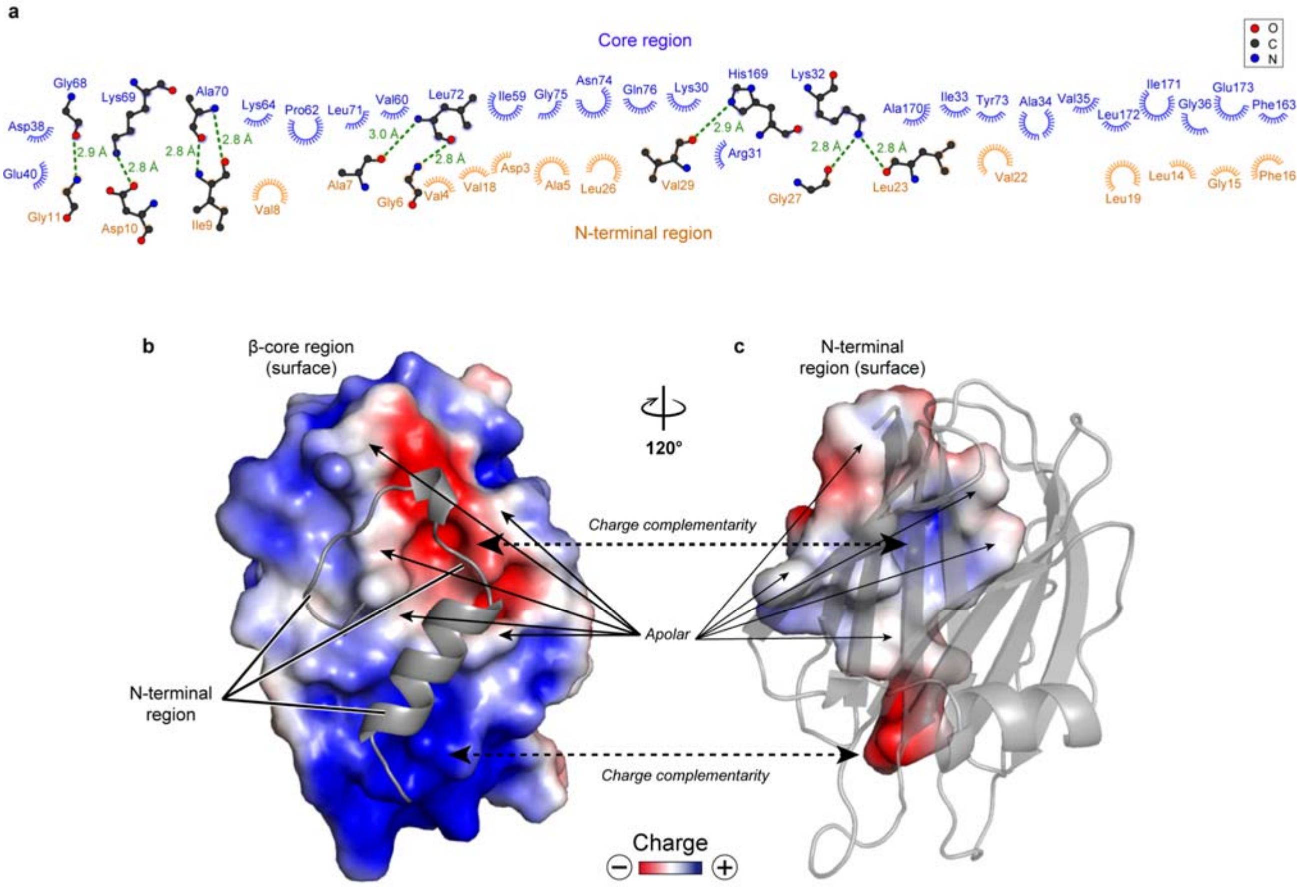

Supplementary Figure 3 | Interactions between the N-terminal region and the β-core region in the monomer. **a,** Interaction between residues of the N-terminal region and the β-core region in the water-soluble monomer. Since the N-terminal region is transferred to the membrane during pore formation, each and every interaction depicted in this panel must be abrogated. Residues belonging to the N-terminal region and the β-core region are depicted in orange and blue, respectively. **b,** Surface representation of the electrostatic potential of the β-core region of the water-soluble FraC monomer (the electrostatic gradient from red to blue represents energies of −3 to +3 kT). The N-terminal region is depicted in cartoon representation (gray). **c,** Electrostatic potential of the N-terminal region (color code is same as above). The β-core region is depicted as cartoon (gray).

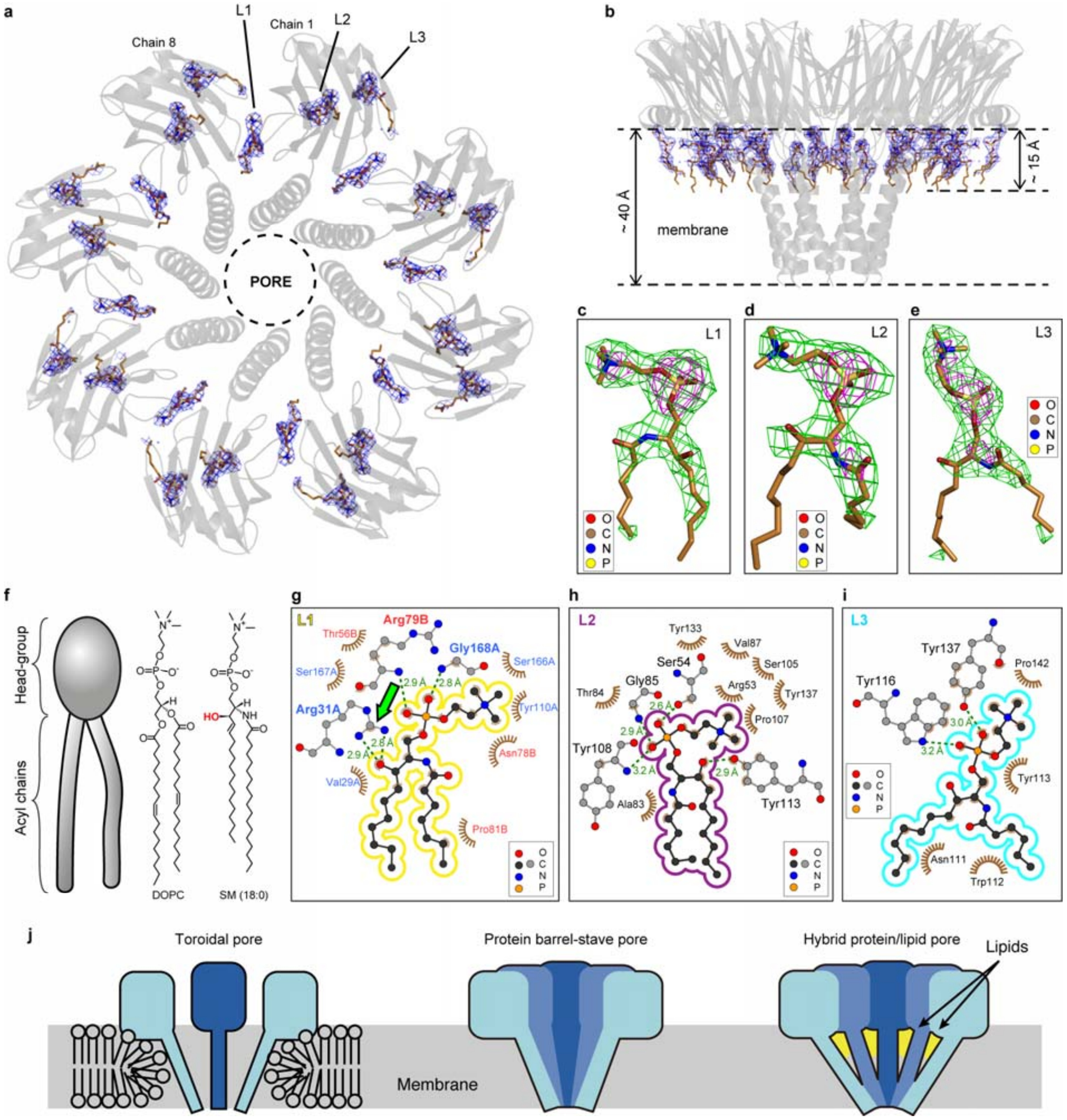

Supplementary Figure 4 | Lipids bound to the pore. a, Top view of the pore of FraC with bound lipids. Lipids are found at three distinct sites. Lipid L1 bridges two protein chains. Lipids L2 and L3 bind to sites at single protein chains. Sigma-A weighted 2Fo − Fc electron density map of the lipids (blue mesh, σ = 1). At the resolution achieved (3.1 Å) is not possible to determine unambiguously if the lipid bound is SM or DOPC, or a mixture of them. SM was chosen (i) because FraC displays higher affinity for SM, and (2) because of the unique ability of SM to make critical H-bonds of functional significance[3]. **b,** Side view of the pore. Lipids align on the same plane mimicking the structure of the outer half of a membrane. **c-e,** Sigma-A weighted difference *omit* electron density map (Fo − Fc) of lipids L1, L2, and L3. Green and purple mesh corresponds to contouring levels of σ = 2.5 and σ = 6.0, respectively. Because of meager features of the electron density maps in the hydrophobic region of the lipids, the length of the acyl-chain of the model was limited to seven carbons. **f,** Scheme of a lipid molecule, and chemical formula of the lipids DOPC (center) and SM 18:0 (right). **g,** Protein environment around lipid L1, **h,** lipid L2, and **i,** lipid L3. **j,** Cross-section of three different models of a pore: Toroidal (left), protein barrel-stave (center) and hybrid protein/lipid (right).

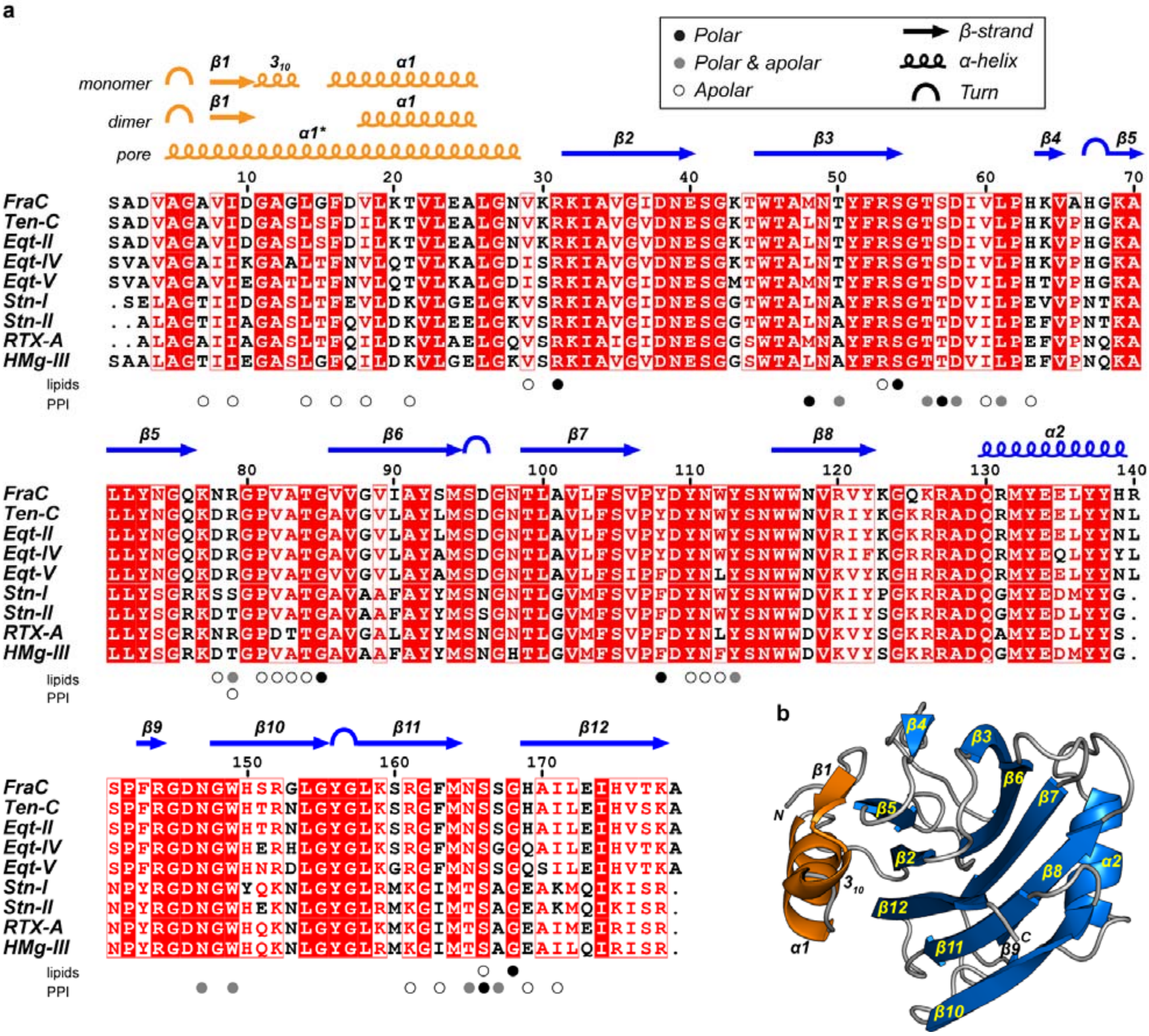

Supplementary Figure 5 | Sequence alignment of actinoporins. a, Sequence alignment of FraC from *Actinia fragacea*[4], tenebrosin-C (Ten-C) from *Actinia tenebrosa*[5]; Eqt-II, equinatoxin IV (Eqt-IV), and equinatoxin V (Eqt-V) from *Actinia equina*[6-8]; sticholysin I (Stn-I) a and sticholysin II (Stn-II) from *Stichodactyla helianthus*[9]; cytolysin RTX-A (RTX-A) from *Radianthus macrodactylus*[10]; and magnificalysin III (HMg-III) from *Heteractis magnifica*[11]. Red background, red text and black text represent the degree of conservation in descending order. The secondary structure elements of the N-terminal region of FraC (monomer, dimer, or pore) are indicated above the sequence. The helix, the arrow, and the inverted U symbols represents α-helix (or 3_{10}-helix), β-strand, and turns, respectively. The secondary structure of FraC is well conserved after residue 30 in the three types of structures of FraC, so only one representation is shown. The alignment was prepared with the software ESPript[12]. Small circles indicate lipid-protein or protein-protein (PPI) interactions. Solid and black, empty, and solid and gray circles represent residues engaging in polar, apolar, or both polar and apolar interactions, respectively. **b,** Structure of monomeric water-soluble FraC illustrates the secondary structure of the toxin. The N-terminal and β-core regions are depicted in blue and orange, respectively.

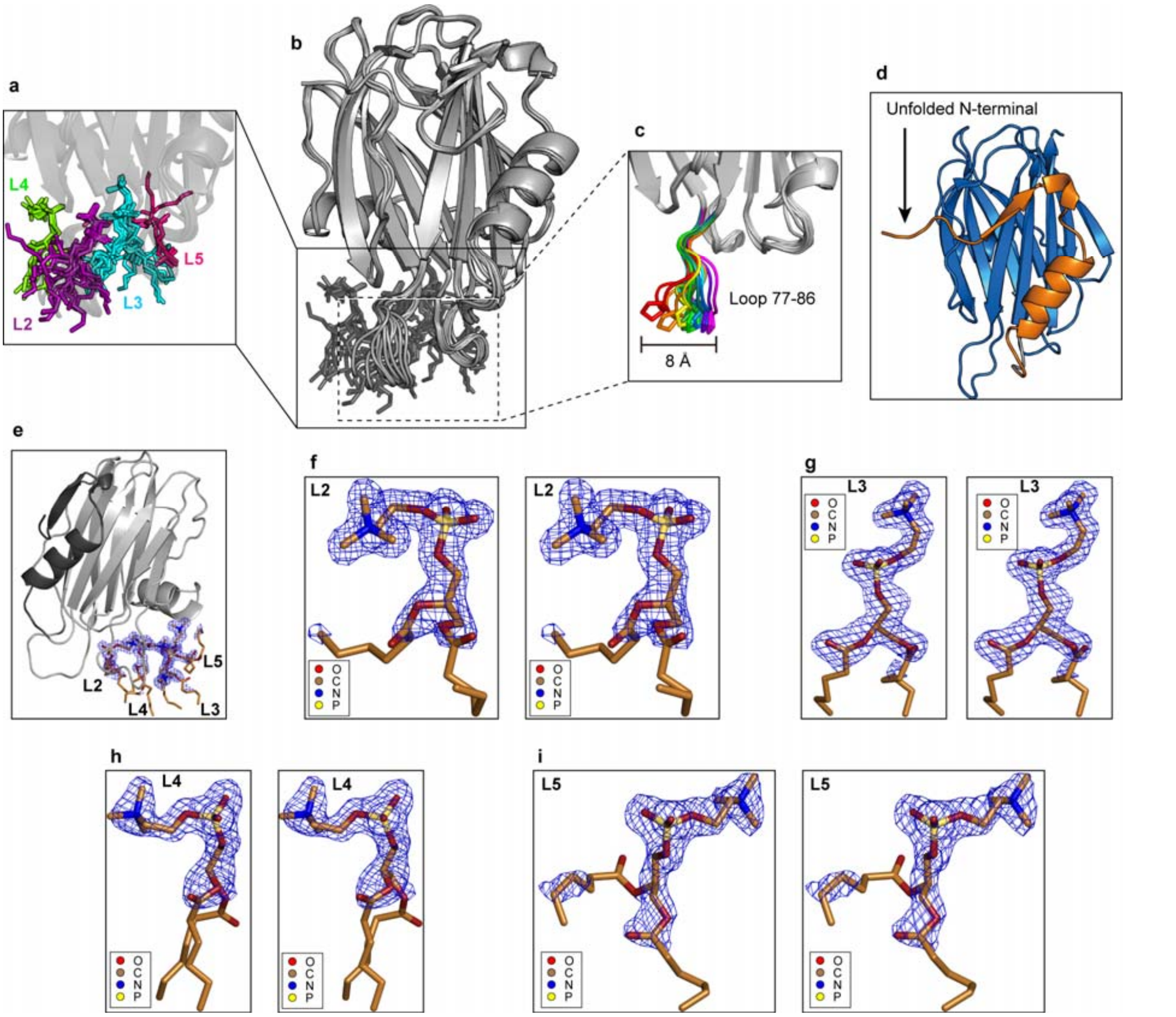

Supplementary Figure 6 | Structure of DHPC bound to monomeric FraC. a-c, Superposition of ten independent crystallographic chains of FraC with DHPC bound (crystallized in three different space groups). **a,** Comparison of all lipids bound to all ten chains of FraC. Lipids termed L2, L3, L4 and L5 are colored in purple, cyan, green and magenta, respectively. **b,** Superposition of all protein chains and lipids. **c,** Close-up view of loop 77-86, which is the element with the highest flexibility in these crystal structures. Notably, the loop 77-86 is the region within the β-core buried the most in the lipid membrane prior to the insertion of the transmembrane helix. **d,** Unfolding of residues Ala2-Gly6 in one of the protein chains of the asymmetric unit. **e,** Sigma-A weighted 2Fo − Fc electron density map contoured at σ = 1 corresponding to the four lipids bound to chain A of FraC (resolution was 1.60 Å). **f-i,** Close-up stereoview of the sigma-A weighted 2Fo − Fc electron density maps of the lipids L2-L5 contoured at σ = 1 (resolution was 1.60 Å).

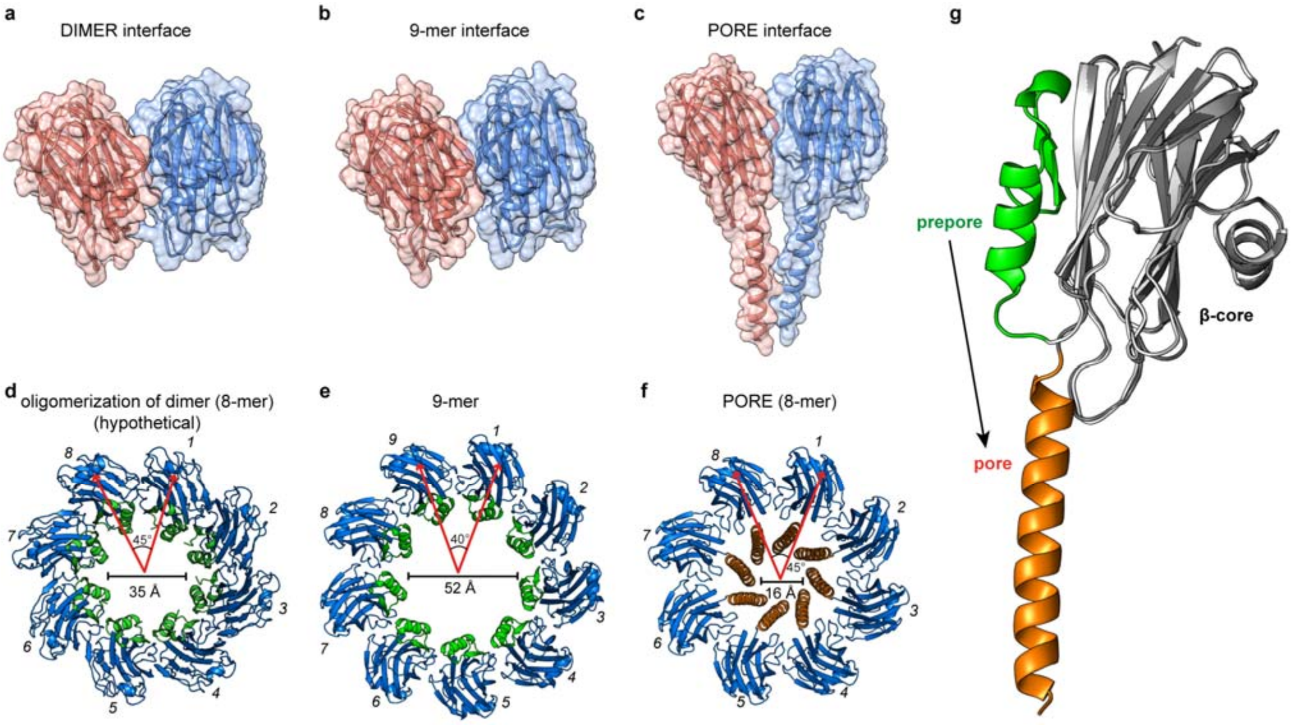

Supplementary Figure 7 | Structural comparison of three oligomeric forms of FraC. a, Protein-protein interface between the two chains of the dimer. **b,** Equivalent interface corresponding to two adjacent chains of the water-soluble structure of 9-mer (PDB entry code 3LIM)[13]. **c,** Equivalent interface of the pore. **d,** Top view of the hypothetical oligomerization of the dimer (same as in Figure 5 of the manuscript). **e,** Top view of the 9-mer (PDB entry code 3LIM). **f,** Equivalent view of the pore of FraC. **g,** Conformational change of FraC from the water-soluble form to the transmembrane pore. The β-core region is shown in light and dark gray for the water-soluble form and the pore, respectively. The N-terminal region is shown in green and orange before and after the metamorphosis, respectively.

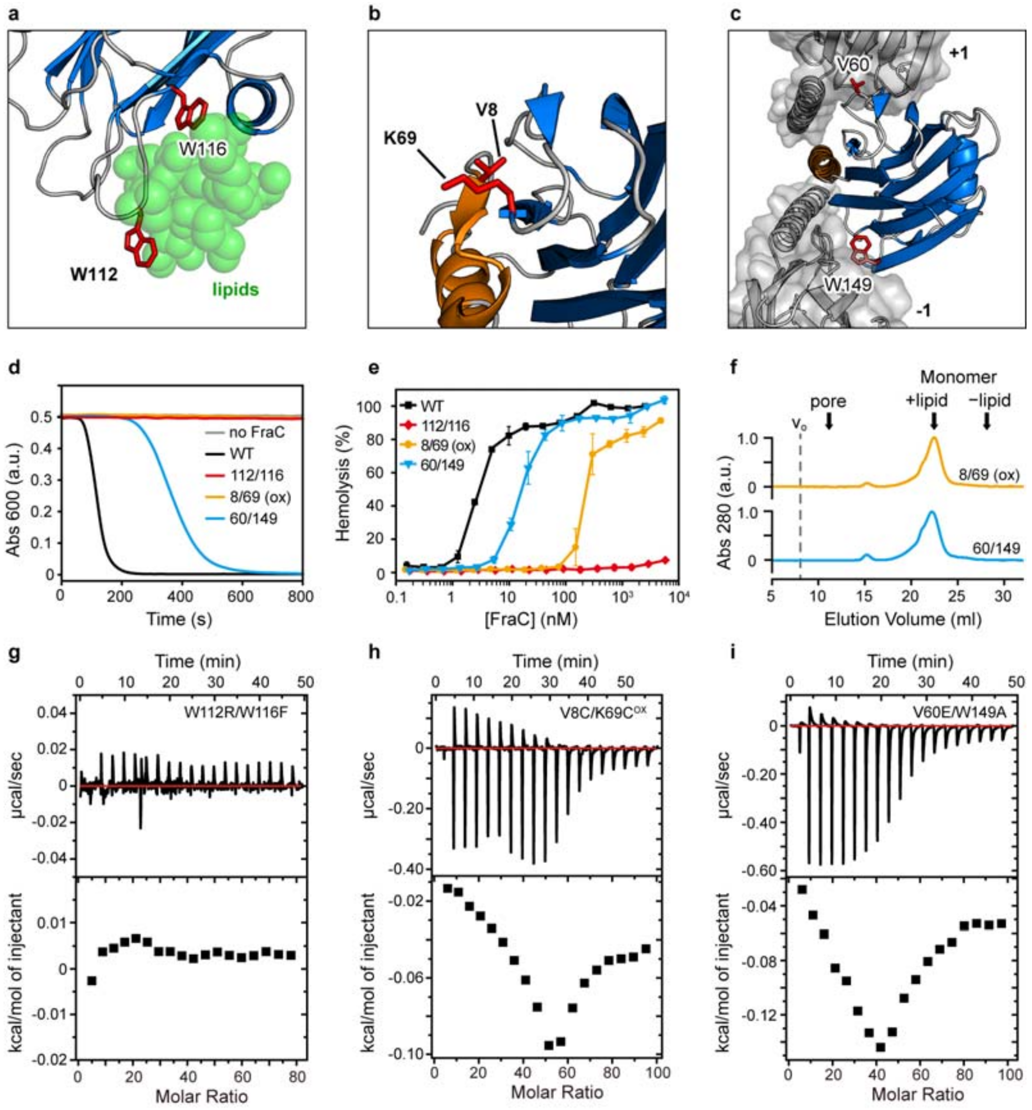

Supplementary Figure 8 | Design, activity and assembly of muteins of FraC. **a,** Location of residues Trp112 and Trp116 in the membrane-binding region. **b,** Position of residues Val8 and Lys69. **c,** Residues Val60 and Trp149 mediate protein-protein interactions between adjacent protomers. These two residues display the largest values of buried surface area (BSA) upon oligomerization (BSA_{VAL60} = 96 $Å^2$; BSA_{TRP149} = 115 $Å^2$). **d,** Kinetic profiles of hemolysis induced by wild-type FraC (black), W112R/W116F (red), V8C/K69C^{OX} (yellow), and V60E/W149A (blue). A control experiment in the absence of FraC is shown in gray. FraC causes cell destruction by a colloid-osmotic mechanism, producing a sharp decrease of turbidity. **e,** Hemolytic activity of wild-type FraC and muteins at equilibrium. Maximum hemolytic activity (100%) was obtained by treating red blood cells with excess wild-type FraC (2.5 µM). **f,** SEC profile of muteins after incubation with vesicles of DOPC/SM (1:1). **g,** Titration of muteins W112R/W116F, **h,** V8C/K69C^{OX}, and **i,** V60E/W149A with liposomes SM/DOPC (molar ratio 1:1) examined by ITC. In panel g no binding is observed. In panels h and i the binding isotherms cannot be explained by a simple binding model.

Supplementary Table 1. Buried surface area (BSA) upon oligomerization (protein-protein).

	Pore	Dimer	9-mer
Residue	BSA (Å)	BSA (Å)	BSA
**Protomer**			
V4		48.1 ± 5.9	28.6 ± 7.2
A5	5.3 ± 1.9	6.9 ± 1.1	11.5 ± 0.4
G6	15.6 ± 2.1	23.4 ± 0.6	14.8 ± 1.1
A7	−	21.2 ± 2.5	−
I9	83.4 ± 1.7	−	−
D10	3.7 ± 1.5	−	−
A12	15.8 ± 0.9	−	−
G13	13.5 ± 1.0	−	−
F16	79.6 ± 1.0	−	−
K20	22.4 ± 1.0	−	−
A47	10.2 ± 0.3	10.3 ± 1.5	11.0 ± 0.5
M48	20.1± 0.2	21.4 ± 6.2	20.1 ± 0.4
N49	20.4 ± 0.9	23.2 ± 2.4	18.1 ± 0.6
T50	25.8 ± 0.5	26.7 ± 0.5	26.2 ± 0.6
F52	16.5 ± 0.4	26.1 ± 0.5	15.5 ± 0.5
T56	26.2 ± 0.2	26.0 ± 3.0	27.8 ± 1.5
S57	24.2 ± 0.3	24.3 ± 1.8	24.7 ± 0.8
D58	50.1 ± 1.1	102.5 ± 6.7	81.8 ± 5.6
I59	23.0 ± 0.4	47.2 ± 1.1	31.3 ± 1.4
V60	95.5 ± 1.2	116.5 ± 0.9	117.1 ± 1.2
L61	26.3 ± 0.5	23.4 ± 0.2	22.1 ± 0.7
H63	77.8 ± 3.1	71.8 ± 5.0	73.3 ± 0.7
Y73	8.5 ± 3.9	3.9 ± 0.2	3.7 ± 0.4
R79	50.1 ± 0.8	73.0 ± 29.3	15.6 ± 4.0
R127	53.5 ± 0.8	46.4 ± 8.7	48.2 ± 1.3
Others	3.4 ± 2.4	29.5 ± 23.3	1.6 ± 0.8
SUM	771 ± 7.0	772 ± 43	593 ± 23
Average[a]	771 ± 8.0	772 ± 43	593 ± 23

	Pore	Dimer	9-mer
Residue	BSA (Å)	BSA (Å)	BSA (Å)
**Protomer +1**			
A7	42.9 ± 1.8	−	−
D10	21.1 ± 0.3	−	−
G11	19.8 ± 1.0	−	−
L14	78.8 ± 1.6	−	−
G15	−	11.6 ± 0.5	−
F16	−	94.1 ± 11.6	107.8 ± 2.9
D17	6.1 ± 0.5	49.1 ± 3.8	24.5 ± 6.8
V18	32.9 ± 0.7	−	−
L19	−	4.1 ± 0.6	−
K20	−	79.2 ± 36.5	17.0 ± 5.2
T21	45.3 ± 1.2	−	−
V22	3.5 ± 0.5	−	−
L23	−	11.7 ± 2.3	5.0 ± 2.2
D146	16.3 ± 0.6	22.2 ± 13.7	10.0 ± 0.4
N147	78.0 ± 1.5	75.1 ± 1.0	81.3 ± 0.9
G148	18.8 ± 0.2	20.0 ± 1.2	20.4 ± 0.4
W149	115.1 ± 5.7	109.8 ± 8.6	112.0 ± 1.3
H150	14.0 ± 0.5	9.7 ± 6.3	16.1 ± 0.7
R161	36.9 ± 1.4	36.4 ± 4.5	39.7 ± 0.5
F163	47.0 ± 0.6	43.5 ± 0.9	52.2 ± 0.4
N165	26.8 ± 0.2	27.0 ± 1.8	31.3 ± 0.6
S166	21.1 ± 0.4	6.3 ± 3.1	13.0 ± 1.6
S167	32.8 ± 0.4	37.1 ± 11.4	25.3 ± 2.1
H169	56.3 ± 0.7	38.5 ± 16.5	7.2 ± 4.2
I171	45.3 ± 0.8	40.7 ± 1.2	18.6 ± 0.7
D173	20.9 ± 0.6	14.0 ± 0.3	13.3 ± 1.0
Others	3.3 ± 2.7	13.6 ± 8.3	
SUM	783 ± 10	743 ± 40	595 ± 20

[a]Average values of protomer and protomer +1.

Supplementary Table 2. H-bonds between protein chains.

Residue 1	Residue 2	Pore	Dimer	9-mer
T50	N147	2.88 ± 0.04	2.95 ± 0.03	3.09 ± 0.02
R127	W149	2.96 ± 0.08	2.80 ± 0.14	2.83 ± 0.04
M48	W149	2.79 ± 0.04	2.84 ± 0.03	2.75 ± 0.03
L61	W149	2.91 ± 0.10	2.91 ± 0.02	2.89 ± 0.04
S57	N165	3.02 ± 0.04	3.12 ± 0.01	−
T56	S166	2.88 ± 0.03	−	−
D58	S167	2.73 ± 0.03	−	−
D58	N165	−	3.16 ± 0.08	−

[a]Distances longer than 3.2 Å are not considered.

Supplementary Table 3. Lipid-protein interactions (H-bonds and BSA) in the structures of the transmembrane pore and the lipid bound form.[a]

LIPID L1			LIPID L2			LIPID L3		
	pore	DHPC		pore	DHPC		pore	DHPC
**H-bonds**								
Res.	Dist. (Å)	Dist. (Å)	Res.	Dist. (Å)	Dist. (Å)	Res.	Dist. (Å)	Dist. (Å)
R31	2.7 ± 0.4	–	S54	2.9 ± 0.2	2.7 ± 0.1	Y137[b]	3.1 ± 0.1	2.6 ± 0.1
R31	3.0 ± 0.1	–	G85	2.7 ± 0.1	2.8 ± 0.05	–	–	–
G168	2.8 ± 0.1	–	Y108[b]	3.1 ± 0.2	2.8 ± 0.04	–	–	–
R79[b]	3.0 ± 0.1	–	Y113[b]	3.0 ± 0.1	–	–	–	–
R79[b]	3.1 ± 0.1	–	–	–	–	–	–	–
R79[b]	3.0 ± 0.1	–	–	–	–	–	–	–
**Interaction surface**								
Res.	BSA (Å^2)	BSA (Å^2)	Res.	BSA (Å^2)	BSA (Å^2)	Res.	BSA (Å^2)	BSA (Å^2)
V29	34 ± 1	–	R53	32 ± 3	43 ± 24	N111	32 ± 4	–
R31	25 ± 1	–	A83	41 ± 7	20 ± 16	W112	100 ± 4	76 ± 14
N78	39 ± 1	–	T84	23 ± 1	29 ± 7	Y113	45 ± 3	44 ± 6
R79	60 ± 1	–	D109	18 ± 4	–	W116	36 ± 1	34 ± 3
P81	38 ± 3	–	W112	30 ± 3	–	Y137	25 ± 1	24 ± 2
V82	26 ± 3	–	Y113	65 ± 3	61 ± 9	Y138	27 ± 2	26 ± 5
Y110	54 ± 1	–	–	–	–	–	–	–
S166	27 ± 1	–	–	–	–	–	–	–
Others	146	–	Others	135	144	Others	54	59
Total	449 ± 12	–	Total	345 ± 17	297 ± 26	Total	320 ± 6	263 ± 18

[a]The structure with the highest resolution and most chains in the asymmetric unit was chosen as representative of the lipid bound-form, designated Lipid-bound (III) in Supplementary Table S1.
[b]Interaction is observed in ≥50% (but not in all) protein chains in the asymmetric unit.

Supplementary Table 4. Interface area (protein-protein) of hemolytic toxins.[a]

	Interface (Å^2)	Oligomer	Pore	Length	PDB Id
**FraC (excluding lipid)**	777 ± 8	8-mer	α-barrel	178	This work
**FraC (including lipid)**	1,226 ± 12	8-mer	α-barrel	178	This work
**ClyA**	2,450 ± 11	12-mer	α-barrel	309	2wcd
**α-hemolysin**	2,766 ± 26	7-mer	β-barrel	293	7ahl
**γ-hemolysin[b]**	2,111 ± 25	8-mer	β-barrel	309 & 290	3b07
**VCC**	2,723 ± 15	7-mer	β-barrel	593	3o44

[a]Interface area was retrieved from the PISA server[14]
[b]γ-hemolysin is composed of two alternating proteins, LukF and Hlg2.

Supplementary Table 5. Residue conservation at the interaction surfaces in the pore.[a,b]

Conservation	Protein-protein	Lipid-protein	All residues
Identical	9	14	95
Strongly conserved	8	3	43
Weakly conserved	4	1	17
Not conserved	2	3	24

[a]The sequences used for the alignment are shown in Supplementary Figure 4.
[b]Conservation was determined with the program ClustalW[15].

Supplementary Table 6. Occupancy of the lipid-binding sites in the three independent crystal structures of FraC with DHPC.[a]

	Lipid site			
Crystal (Chain)	L2	L3	L4	L5
$P3_2$ (A)	++	++	++	++
$P3_2$ (B)	++	++	++	++
$P3_2$ (C)	++	++	+	−
$P3_2$ (D)	++	++	+	−
$P3_2$ (E)	++	++	+	+
$P3_2$ (F)	+	++	+	−
P321 (A)	++	++	++	−
P321 (B)	++	++	−	−
$P4_22_12$ (A)	++	++	−	−
$P4_22_12$ (B)	++	++	−	−
Total[b]	10 (9)	10 (10)	7 (3)	3 (2)

[a]Modeling of DHPC and POC (in some cases the e-density of the glycerol and acyl chain is absent, but not that of the POC moiety) is indicated with the symbols (++) and (+), respectively. Absence of lipid and POC is indicated with the symbol (−).
[b]Values in parenthesis indicate the number of DHPC molecules.

SUPPLEMENTARY REFERENCES

1 Wohri, A. B. *et al.* A lipidic-sponge phase screen for membrane protein crystallization. *Structure* **16**, 1003-1009, (2008).

2 Veesler, D. *et al.* Production and biophysical characterization of the CorA transporter from Methanosarcina mazei. *Anal Biochem* **388**, 115-121, (2009).

3 Maula, T. *et al.* 2NH and 3OH are crucial structural requirements in sphingomyelin for sticholysin II binding and pore formation in bilayer membranes. *Biochim. Biophys. Acta* **1828**, 1390-1395, (2013).

4 Bellomio, A. *et al.* Purification, cloning and characterization of fragaceatoxin C, a novel actinoporin from the sea anemone *Actinia fragacea*. *Toxicon* **54**, 869-880, (2009).

5 Simpson, R. J., Reid, G. E., Moritz, R. L., Morton, C. & Norton, R. S. Complete Amino-Acid-Sequence of Tenebrosin-C, a Cardiac Stimulatory and Hemolytic Protein from the Sea-Anemone Actinia-Tenebrosa. *Eur. J. Biochem.* **190**, 319-328, (1990).

6 Anderluh, G. *et al.* Equinatoxins, pore-forming proteins from the sea anemone Actinia equina, belong to a multigene family. *Toxicon* **37**, 1391-1401, (1999).

7 Belmonte, G. *et al.* Primary and Secondary Structure of a Pore-Forming Toxin from the Sea-Anemone, Actinia-Equina L, and Its Association with Lipid Vesicles. *Biochim. Biophys. Acta* **1192**, 197-204, (1994).

8 Pungercar, J., Anderluh, G., Macek, P., Franc, G. & Strukelj, B. Sequence analysis of the cDNA encoding the precursor of equinatoxin V, a newly discovered hemolysin from the sea anemone Actinia equina. *Biochim. Biophys. Acta* **1341**, 105-107, (1997).

9 Huerta, V. *et al.* Primacy structure of two cytolysin isoforms from Stichodactyla helianthus differing in their hemolytic activity. *Toxicon* **39**, 1253-1256, (2001).

10 Il'ina, A. *et al.* Amino acid sequence of RTX-A's isoform actinoporin from the sea anemone, Radianthus macrodactylus. *Toxicon* **47**, 517-520, (2006).

11 Wang, Y. W., Chua, K. L. & Khoo, H. E. A new cytolysin from the sea anemone, Heteractis magnifica: isolation, cDNA cloning and functional expression. *Biochim. Biophys. Acta* **1478**, 9-18, (2000).

12 Gouet, P., Robert, X. & Courcelle, E. ESPript/ENDscript: extracting and rendering sequence and 3D information from atomic structures of proteins. *Nucleic Acids Res.* **31**, 3320-3323, (2003).

13 Mechaly, A. E. *et al.* Structural insights into the oligomerization and architecture of eukaryotic membrane pore-forming toxins. *Structure* **19**, 181-191, (2011).

14 Krissinel, E. & Henrick, K. Inference of macromolecular assemblies from crystalline state. *J. Mol. Biol.* **372**, 774-797, (2007).

15 Larkin, M. A. *et al.* Clustal W and clustal X version 2.0. *Bioinformatics* **23**, 2947-2948, (2007).

16 Pettersen, E. F. *et al.* UCSF Chimera--a visualization system for exploratory research and analysis. *J Comput Chem* **25**, 1605-1612, (2004).
